# Experimental simulation of environmental warming selects against pigmented morphs of land snails

**DOI:** 10.1002/ece3.7002

**Published:** 2021-01-14

**Authors:** Heinz‐R. Köhler, Yvan Capowiez, Christophe Mazzia, Helene Eckstein, Nils Kaczmarek, Mark C. Bilton, Janne K. Y. Burmester, Line Capowiez, Luis J. Chueca, Leonardo Favilli, Josep Florit Gomila, Giuseppe Manganelli, Silvia Mazzuca, Gregorio Moreno‐Rueda, Katharina Peschke, Amalia Piro, Josep Quintana Cardona, Lilith Sawallich, Alexandra E. Staikou, Henri A. Thomassen, Rita Triebskorn

**Affiliations:** ^1^ Animal Physiological Ecology Institute for Evolution and Ecology University of Tübingen Tübingen Germany; ^2^ INRA, UMR 1114 Site Agroparc Avignon Cedex 9 France; ^3^ Mediterranean Institute of Marine and Terrestrial Biodiversity and Ecology (IMBE) UMR 7263 AMU, CNRS Université d´Avignon Avignon Cedex 9 France; ^4^ Namibian University of Science and Technology Windhoek Namibia; ^5^ Senckenberg Biodiversity and Climate Research Centre Frankfurt am Main Germany; ^6^ Department of Zoology and Animal Cell Biology Faculty of Pharmacy University of the Basque Country (UPV/EHU) Vitoria‐Gasteiz Spain; ^7^ Dipartimento di Scienze Fisiche, della Terra e dell'Ambiente Sezione di Scienze Ambientali Università degli Studi di Siena Siena Italy; ^8^ Maó Illes Balears Spain; ^9^ Lab of Plant Biology and Plant Proteomics Department of Chemistry and Chemical Technologies University of Calabria Rende Italy; ^10^ Departamento de Zoología Facultad de Ciencias Universidad de Granada Granada Spain; ^11^ Institut Català de Paleontologia Miquel Crusafont Universitat Autònoma de Barcelona Edifici ICTA‐ICP, campus de la UAB Barcelona Spain; ^12^ Ciutadella de Menorca Illes Balears Spain; ^13^ Department of Zoology School of Biology Aristotle University of Thessaloniki Thessaloniki Greece; ^14^ Comparative Zoology Institute for Evolution and Ecology University of Tübingen Tübingen Germany; ^15^ Steinbeis‐Transfer Centre for Ecotoxicology and Ecophysiology Rottenburg Germany

**Keywords:** global change, oxidative stress, radiation, shell color, stress proteins, thermal selection

## Abstract

In terrestrial snails, thermal selection acts on shell coloration. However, the biological relevance of small differences in the intensity of shell pigmentation and the associated thermodynamic, physiological, and evolutionary consequences for snail diversity within the course of environmental warming are still insufficiently understood. To relate temperature‐driven internal heating, protein and membrane integrity impairment, escape behavior, place of residence selection, water loss, and mortality, we used experimentally warmed open‐top chambers and field observations with a total of >11,000 naturally or experimentally colored individuals of the highly polymorphic species *Theba pisana* (O.F. MÜller, 1774). We show that solar radiation in their natural Mediterranean habitat in Southern France poses intensifying thermal stress on increasingly pigmented snails that cannot be compensated for by behavioral responses. Individuals of all morphs acted neither jointly nor actively competed in climbing behavior, but acted similarly regardless of neighbor pigmentation intensity. Consequently, dark morphs progressively suffered from high internal temperatures, oxidative stress, and a breakdown of the chaperone system. Concomitant with increasing water loss, mortality increased with more intense pigmentation under simulated global warming conditions. In parallel with an increase in mean ambient temperature of 1.34°C over the past 30 years, the mortality rate of pigmented individuals in the field is, currently, about 50% higher than that of white morphs. A further increase of 1.12°C, as experimentally simulated in our study, would elevate this rate by another 26%. For 34 *T. pisana* populations from locations that are up to 2.7°C warmer than our experimental site, we show that both the frequency of pigmented morphs and overall pigmentation intensity decrease with an increase in average summer temperatures. We therefore predict a continuing strong decline in the frequency of pigmented morphs and a decrease in overall pigmentation intensity with ongoing global change in areas with strong solar radiation.

## Graphical abstract

We simulated environmental warming for 1.12°C in the Mediterranean, and related temperature‐driven internal heating, protein and membrane integrity impairment, escape behavior, place of residence selection, water loss, and mortality in a total of >11,000 naturally or experimentally colored individuals of the highly polymorphic land snail species *Theba pisana*. These molecular and physiological responses lead to elevated mortality in naturally pigmented morphs by 26%. By comparing the situation in 35 Mediterranean T. pisana populations, we show that both the frequency of pigmented morphs and overall pigmentation intensity decrease with an increase in average summer temperatures.

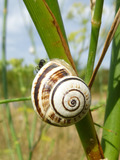

## 1 INTRODUCTION

To better understand and predict the impact of climate warming on organisms requires multitrait approaches (Debecker & Stoks, 2019). Recognizing the potential impacts of global warming, the physiological mechanisms that limit heat tolerance in ectotherms regained interest around the turn of the millennium (Pörtner, 2002). More recently, the entirety of phenotypic traits including behavior, physiology, and life cycle parameters that correlate in concert with environmental variation, has been identified and studied as syndromes (Boyle et al., 2015; Killen et al., 2013), helping to gain further understanding into the mechanisms behind the selection pressures generated by climate change (Brook et al., 2008; Stevens et al., 2013). Among the best‐studied examples for phenotypic variation in response to climate and habitat are shell pigmentation polymorphisms of helicoid land snails, which have been investigated in detail for more than 50 years (reviewed in Ożgo, 2014; Schilthuizen, 2012; Schweizer et al., 2019). Numerous studies on these animals have addressed single morphological features, physiological traits, or biochemical parameters that are modified by thermal stress. However, the concurrent syndromic responses to thermal stress have rarely been addressed, but are of crucial importance for a mechanistic understanding of stress responses across biological levels of increasing complexity (Schweizer et al., 2019).

The evolutionary consequences of fluctuating climate for the frequency of differently pigmented morphs of land snails have been investigated in a number of extensive studies. Despite interannual frequency changes, a high variation in shell pigmentation was found to be maintained over decades (Cain, 1983; Cain et al., 1990; Cameron, 1992, 2001; Cameron & Pokryszko, 2008; Cook et al., 1999; Cook & Pettitt, 1998; Cowie, 1992; Cowie & Jones, 1998; Johnson, 2011; Murray & Clarke, 1978; Ożgo & Kinnison, 2008; Silvertown et al., 2011; Wall et al., 1980). Changes in pigmentation intensity were interpreted to result from temporal and spatial variation in the strength and direction of selection (Cook, 2005; Johnson, 2011, 2012) or from epigenetic nondirectional changes within the limits of phenotypic plasticity (Köhler et al., 2009, 2013). Despite their unquestionable importance, none of these studies comprised experimentally modified field conditions or measured physiological or biochemical parameters that could have helped to mechanistically understand thermal stress‐exerted selection pressure. Nevertheless, other studies that correlated habitat characteristics with morph frequencies found shells with a high albedo to be advantageous under high solar radiation, for example, in open habitats (Cowie, 1990; Cowie & Jones, 1985; Currey & Cain, 1968; Jones et al., 1977; Ożgo, 2011; Ożgo & Bogucki, 2011; Ożgo & Kinnison, 2008; Ożgo & Komorowska, 2009; Richards & Murray, 1975; Schilthuizen, 2013). These results were supported by heating experiments with naturally or artificially pigmented shells (Cook & Freeman, 1986; Dieterich et al., 2015; Hazel & Johnson, 1990; Heath, 1975; Jones, 1973; Moreno‐Rueda, 2008) and a field study that involved physiological and behavioral parameters (Staikou, 1999).

In addition, two resurvey studies in England and the Netherlands revealed an increase in individuals with high‐albedo shells at the cost of darker ones after 43 years in the garden snail, *Cepaea nemoralis* (Cameron et al., 2013; Ożgo & Schilthuizen, 2011), which was interpreted to be adaptive to environmental temperature increase. Stine (1989) reported the same effect for *C. nemoralis* after several decades for Virginia, USA, Johnson (2011) for *Theba pisana* in Western Australia. Another large study that specifically addressed the effects of climate change‐induced temperature increases in Europe revealed an increase in yellow individuals in sand dunes, the most exposed habitat type, but did not find general support for selective advantages of high shell albedo across habitats in *C. nemoralis* (Silvertown et al., 2011). Although land surface temperatures in Europe have increased by an average 1.3°C during the 20th century until 2009 (European Environment Agency, 2018), data obtained in this study made evident that garden snails did not show any increase in the frequency of the lightest (yellow) morphs over time but rather an unexpected decrease in the frequency of unbanded shells (independent of the basic coloration, which can be yellow, pink, or brown) and an increase in the “mid‐banded” morph. Although precise shell pigmentation intensity had not been quantified, Silvertown et al. (2011) and Cameron and Cook (2012) concluded that banding was probably not the most important trait that selection had acted upon. They suggested that factors other than climate change‐induced temperature increases, such as changing predation pressure or habitat changes affecting microclimatic conditions, might have asserted stronger selection pressure. Indeed, many other studies have suggested alternative explanations to thermal selection for variation in the banding frequency of land snails. For example, the selection on visual crypsis through predation by birds or rodents has often been hypothesized as a key component limiting morph frequencies (Allen, 2004; Bond, 2007; Cain & Currey, 1963; Cain & Sheppard, 1954; Clarke, 1969; Cook, 2005; Endler, 1978; Heller & Gadot, 1984; Moreno‐Rueda, 2009; Punzalan et al., 2005; Rosin et al., 2011). Furthermore, while a recent study on *C. nemoralis* from urban “heat island” areas in the Netherlands found a higher frequency of the palest morph (yellow), these yellow snails were also more likely to carry bands on the umbilical side of the shell. As a result, the authors proposed a possible role of such pigmentation in a yet unknown way (Kerstes et al., 2019), adding further complexity to the theory of selection due to thermal stress.

The manifold results and conclusions of these studies suggest that the role of thermal selection in the evolutionary ecology of land snails in response to global change, and its relevance for differently pigmented morphs, remains only partly understood. This is especially true in snails with extremely variable shell coloration such as the Mediterranean species *T. pisana*. In this species, shell color and banding patterns are primarily determined by alleles of at least three loci (Cain, 1984; Cowie, 1984), but the interplay of genetic variation and phenotypic plasticity results in a situation in which almost every individual displays a unique pigmentation pattern and intensity (Cain, 1984; Cowie, 1984; Johnson, 2012; Köhler et al., 2013). Furthermore, physical integrity and survival over the hot season are of particular evolutionary relevance in *T. pisana* because this species reproduces at the end of its lifetime. In this context, combining field observations with common garden experiments, the analysis of trait syndromes can shed light on the causal effects of thermal selection. The Mediterranean basin is globally among the regions which are most vulnerable to climate change, with annual mean surface air temperature increasing from 0.19 to 0.25°C per decade between 1960 and 2005 (Mariotti et al., 2015) and 1.1°C from 1985 to 2015 (Cramer et al., 2018). Depending on the climate scenario and the season, a rise in temperature versus pre‐industrial time from 2 to 6°C by 2100 is expected in the Mediterranean (Giorgi & Lionelli, 2008; Jacob et al., 2014). Using *T. pisana*, we here experimentally simulated warming in the expected range under global climate change and artificially altered shell pigmentation in a Mediterranean field plot. In a multitrait approach, we combined our experiments with field measurements of thermodynamics, behavioral studies on snail competition, biochemical markers for protein and lipid destruction, and measurements of transpiration and survival. The goal of this study thus was to identify a mechanistically plausible interplay between thermodynamics, biochemistry, physiology, and anticipated population changes in the course of global warming at a given site in one of the prospectively most threatened regions on earth. Connecting field measurements with passive open‐top chambers (OTCs) to manipulate air and soil surface temperatures (Henry & Molau, 1997; Marion et al., 1997; Welshofer et al., 2018; Yan et al., 2015), we aimed to test the following hypotheses:

- [H1] Even faint differences in shell pigmentation intensity result in different warming of morphs.
- [H2] Simulation of global warming causes increasing protein degradation and oxidative stress in increasingly pigmented snails.
- [H3] As *T. pisana* often forms clusters at resting sites, morphs of different pigmentation intensity interact (i.e., actively compete or act jointly) in their behavior when escaping the hot soil surface in a morph‐specific way.
- [H4] Water loss due to environmental warming differs among different morphs.
- [H5] Survival rates of morphs decrease with increasing pigmentation intensity under simulated global warming conditions.

The use of OTCs enabled us to change the expression of fluctuations in environmental temperature rather than just to increase a constant mean temperature. This approach thus allowed close to nature acclimation independent of the basal thermotolerance (Gerken et al., 2015) and avoided shifts in the animals' performance optima that may occur under constant conditions (Dowd et al., 2015). Using our OTC‐derived data, we also aimed to predict future morph frequencies and average shell pigmentation intensity in our experimental field site with ongoing global warming. We tested our predictions by field observations on morph frequencies in other Mediterranean sites that already are subject to such thermal conditions that are expected for the experimental site.

## 2 MATERIALS AND METHODS

### 2.1 Field site and population abundance

**Figure 1:**
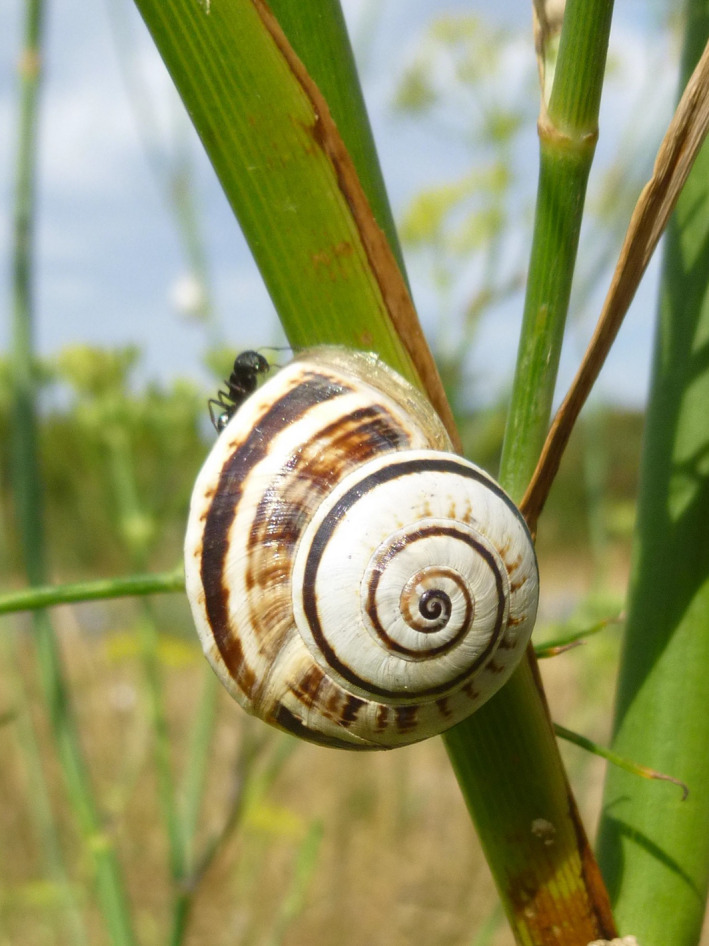
*Theba pisana* in its natural habitat at Montfavet, Southern France

For the main part of the experiments, we selected a site close to Montfavet (43°54.984′N, 4°53.772′E) in the Avignon area of Southern France with a high abundance of *T. pisana* (O.F. MÜller, 1774) (Figure 1). The site is in the vicinity of Avignon climate station from which we obtained climate data (global solar irradiation, air temperature in the shade at 1 m above the ground, and precipitation) for the time of our experiments (9 August–6 September 2017; temporal resolution 15 min). Furthermore, we obtained long‐term temperature data (monthly maximum, monthly mean) for this site over a 30‐year period (January 1988–December 2017, resolution 1 month). Individuals of *T. pisana* from the field site were classified according to the pigmentation of their shells in two categories, “naturally white” (W) and “naturally pigmented” (P), the latter comprising individuals with one or more (even faint or dotted) bands. To quantify the frequency of both morphs at the field site, 24 samples of randomly collected snails with a total number of 3,339 individuals were taken from the vegetation and the snails allocated to the categories W and P. The scarcity of vegetation at this site prevented potential camouflage of snails and thus bias in sampling, as all individuals were easily visible. We refrained from sampling empty shells from the ground to circumvent uncertainty in respect to *postmortem* shell bleaching. To quantify the mortality of the different morphs in the field under natural conditions, we randomly collected 2,160 individuals that were attached to the vegetation by dried mucus surrounding the aperture, allocated them to the categories W and P, and checked whether individuals were dead or alive.

### 2.2 Manipulation of snail pigmentation

The purpose of shell color manipulation was to provide a series of morphs with gradually increasing pigmentation intensity from white to black. As most individuals at the Montfavet site were naturally white, we exclusively applied black ink on them and refrained from painting pigmented ones white. All experiments were thus conducted with four morphs of *T. pisana*. Apart from the two natural morph classes (“naturally white,” W; and “naturally pigmented,” P), we experimentally colored white individuals either by adding a rather thick band to the apical side of the shell (“artificially striped,” S) or by painting both sides of the shell entirely black (“artificially blackened,” B) using a carbon‐based tattoo ink on water basis without solvents and free from heavy metals, phenol, and polycyclic aromatic hydrocarbons (Deep Colours! Black, DC‐TP Europe LLC). Using scanning electron microscopy (SEM), we found that the artificial black color only added a very thin layer of pigment to the exterior of the shell. The fine structure of the shell's surface was smoothened in the painted areas. This smoothening, however, rather resembles the natural surface of pigmented shell parts (bands) in *T. pisana*, as bands exhibit a smoother surface than pale shell parts in SEM pictures (Schweizer et al., 2019).

### 2.3 Escape behavior and snail temperature

For the studies on escape behavior, we alternately placed five individuals of two morphs per run (six possible morph combinations) in a row on the soil surface in front of a 20‐cm high wall of an open‐top transparent polyethylene frame with a ground area of 30 × 20 cm, a so‐called “open‐top chamber.” Thirty individuals per morph, that is, 120 individuals, were tested on three different days each, resulting in 360 behavioral recordings. All morph combinations were run in an equal number under the same conditions, following a random sequence. During the experiments, the soil surface temperature was 55.3 ± 3.7°C, insolation was 84.6 ± 3.3 klx, and the air temperature at 1 m above ground level was 33.6 ± 3.1°C. In each run, individuals were ranked according to the time they needed to (a) move on the hot soil surface, (b) start climbing the open‐top chamber's wall, and (c) reach 20 cm height to the wall's top (rank 1 quickest, rank 10 slowest). Rank sums were calculated for each morph and each behavioral criterion (a–c). Subsequently, for each morph pair, a Δ rank sum was calculated (the rank sum of the darker morph minus the rank sum of the lighter morph), which was related to the difference in pigmentation intensity calculated for the corresponding pair of snails (for pigmentation measurements see below). We recorded randomly selected runs with a high‐resolution thermocamera (Micro‐Epsilon Thermo Imager 400, equipped with a macrolens) and measured shell and soft body temperature of the snails on the ground (*n* = 31 for each morph) and at heights of 6.5 cm (*n* = 18–25), 10 cm (*n* = 17–21), and 20 cm (*n* = 14–17) above ground level for every individual possible.

### 2.4 Morph‐specific body and shell temperatures

Individuals from all four morphs were kept in the laboratory at 22°C in a box, which was transported outside right before measuring body and shell temperatures. Two individuals from different morphs were randomly placed within 30 s on two needle thermometers that had been calibrated against one another at a height of 50 cm above ground in the full sunlight (90–100 klux). After piercing the body at the aperture, next to the parietal wall with the needle, the internal temperatures of the two individuals were measured at 30‐ to 60‐s intervals in the sun. At the same time, the shell temperature (apical side) was measured using high‐resolution thermography. We calculated ΔT (T of the darker morph minus T of the lighter morph) for both the internal and shell temperatures, and for each pair of snails. In total, 44 individuals were measured. All snails survived the piercing of their skin.

### 2.5 Open‐top chamber experiments on climbing ability, water loss, and mortality

Passive open‐top chambers were constructed for the purpose of locally raising the temperature in the snails’ habitat, simulating the conditions of environmental warming (Marion et al., 1997; Welshofer et al., 2018). Fifty‐four open‐top transparent polyethylene chambers (OTCs) with a ground area of 30 × 20 cm and a height of 20 cm were arranged in a 9 × 6 block design. OTCs were positioned on the ground of a freshly mowed area of natural grassland on the INRA Avignon territory, 50 m away from Avignon climate station, and fixed with wire and spikes on the outside. The experimental area was 12 m × 12 m, level, and shadeless. Prior to the experiment, all abundant land snails (predominantly *T. pisana* and *Xeropicta derbentina*) were removed from the experimental area. To offer climbing facilities for snails, in every OTC two rows of each five wooden skewers (10 in total) were vertically inserted into the ground, leaving a length of 25 cm above the ground surface. To avoid the escape of snails, the upper rim of each OTC was lubricated with a lemon extract‐containing paste (IRKA Meitingen) which repelled snails, but had previously been shown not to cause mortality or elevated weight loss (own unpublished data, not shown).

The experiment was designed to investigate (a) the survival strategies of individual snails and (b) potential competition or joint action between them. To do so, in each of the nine blocks, all six possible binary combinations of the four morphs were tested in one OTC. Always 20 individuals from two morphs—that is, 40 snails per OTC—were introduced into the OTCs on 9 August 2017. The experiment lasted until 4 September 2017. Climate data for this time span were obtained from Avignon climate station (global solar irradiation [J/cm^2^], air temperature at 1 m height [°C], rainfall [mm]) and from two data loggers inside and outside a reference OTC from 18 August to 6 September 2017 (air temperature at 5 cm above ground), showing that this experiment simulated environmental warming of 1.12 ± 0.94°C above ambient temperatures. Control snails were kept in the laboratory at a constant temperature of 25°C and a natural day/night regime.

Climbing behavior and mortality were recorded in each OTC on days 1, 2, 3, 5, 7, 9, 12, 14, 16, 19, 21, 23, and 26. For climbing, we counted the number of morphs that had climbed ≥10 cm on a skewer, as well as the number and identity of the morph that had climbed highest, but at least 10 cm (“top” position). After counting, all individuals were placed on the ground again to avoid inactivity (and thus double counting) at an elevated position for more than one day. Mortality (i.e., the number of dead snails relative to the starting number) was recorded on the same days for each OTC and each morph. All snails were weighed at day 0 (prior to the introduction into the OTCs) and at day 8. As it was impossible to mark the snails individually, we concentrated on the average fresh weight of surviving snails for every morph and OTC.

Climbing, mortality, and weight measurements were statistically analyzed using generalized liner mixed models in R version 3.4.2 (R Development Core Team, 2014) using package “lme4” (Bates et al., 2015). Statistical significance was calculated by model simplification, with multicomparison tests and estimates performed using least‐square means (emmeans package, Lenth, 2018). Intercepts of OTC identity were used as random variables in all models.

Climbing had three response variables: (a) “number of a morph higher than 10 cm” per OTC—modeled as a either a Poisson distribution (log‐link function) with the number of individuals per morph that climbed higher than 10 cm, or modeled as (b) a binomial distribution (logit link function) with the ratio of individuals per time point per OTC above 10 cm out of the number of living individuals per morph per time point and (c) “number of top positions occupied” per OTC—modeled as a binominal distribution (logit link function) with the ratio of individuals taking up top positions out of the number of skewers (10) or number of living individuals per morph if < 10.

We also used two response variables for mortality: “Death rate”—modeled as a binomial distribution (logit link function), with the number of living individuals out of the total starting number (20)—and “time to D50”—modeled as a gamma distribution (log‐link function) as the time (days) it took for 50% (10 individuals per morph) to die (note that if 10 deaths were reached between observational recordings, fractional days were calculated on a linear scale).

For both climbing and mortality, models were constructed to investigate OTCs that only contained the individual pairwise comparisons of morph type (i.e., B vs. W or B vs. S) “single‐competition” OTCs. Models were also constructed to investigate overall comparisons of morph type acting as either the “competitive response” of an individual morph (the value of the response variable being dependent on the morph of its neighbor) or “competitive effect” of an individual morph (the value of the neighboring morph in response to any impact a morph may have). Tests were made for each morph to test for differences in the effect or response with all three alternative morph combinations. General patterns were also calculated for competitive response and effect using like‐for‐like comparisons, for example, in a comparison of the competitive responses of B versus W morphs, we also included B versus S and B versus P comparisons to W versus S and W versus P, in order to remove bias from W versus B OTCs and B versus W OTCs.

To test for differences in morph response and effect on climbing, solely early observational time points at 2, 3, and 5 days were used with a categorical explanatory variable “time” included in the model, to exclude impact of death at later time points from the model. Death rate also included time as a continuous variable from points 1 to 9, as later time points included too many zeros for a reliable analysis. Here, the slope and therefore interaction of morph identity x time were the important factor of analysis. Finally, weight at time 0 and time point 8 were analyzed separately to determine differences in weight dependent on morph type. Weights were log‐transformed to satisfy model assumptions, with OTC identity included as a random variable.

### 2.6 OTC experiments on snail temperature, oxidative stress, and proteotoxicity

Sixteen open‐top transparent polyethylene chambers (OTCs) with a ground area of 30 × 20 cm and a height of 20 cm were arranged in a 4 × 4 block design on the field ground mentioned before (INRA Avignon territory, 3 blocks) and in the laboratory (control at 25°C, 1 block). The OTCs were equipped as described above. In each of the four blocks, the four OTCs housed a single morph (W, P, S, or B) with 20 individuals. Animals were exposed to ambient conditions from 4 September 2017 to 6 September 2017, and mortality was recorded. At the end of the exposure, we measured shell temperature by thermography and, after freezing, levels of oxidative stress and proteotoxicity of the surviving individuals as follows. At the same afternoon of 6 September, we sampled 40 individuals of each W and P abundant at the Montfavet field site and froze them in liquid nitrogen for oxidative stress (*n* = 20) and proteotoxicity analysis (*n* = 20).

Thermography: Thermographic pictures of the three field‐installed blocks of OTCs were taken by a Micro‐Epsilon Thermo Imager 400, and the shell temperature of those individuals that have been climbing up the wooden skewers was morph‐specifically quantified for every individual possible (W: *n* = 40, P: *n* = 51, S: *n* = 41, B: *n* = 30).

Oxidative stress: Lipid peroxidation was quantified according to the method of Troschinski et al. (2014), which is a modification of the original method (“FOX assay”) of Hermes‐Lima et al. (1995). Individuals were weighed and homogenized in ice‐cold HPLC grade methanol (dilution 1:5; the required amount of methanol is calculated by the division of wet mass of the individual by density of methanol: 0.791 g/cm^3^), and centrifuged at 15,000 *g* and 4°C for 5′. Supernatants were stored at –80°C until further analysis. The assay was conducted in 96‐well plates. In each well (except for the blank), 50 μl of each reagent was added in the following order: 0.25 mM FeSO_4_, 25 mM H_2_SO_4,_ and 0.1 mM xylenol orange. Then, 40 μl of sample supernatant was added and the final sample volume adjusted to 200 μl with aqua bidest. For each sample, three replicate wells were measured and the mean was calculated. Master blanks contained 200 μl of aqua bidest. Samples were incubated at room temperature for 180 min, and absorbance was then read at 580 nm (*A*_580_) using an automated microplate photospectrometer (Elx8006; Bio Tek Instruments). Then, 1 μl of 1 mM CHP solution was added to the samples, incubated for 30 min at room temperature, and again read at 580 nm (*A*_580+CHP_). The content of lipid hydroperoxides in the samples is expressed as CHP equivalents per gram wet mass (CHPE/g wet mass) and was calculated according to the equation (Hermes‐Lima et al., 1995).

$$\text{CHPE/g}=\left(A_{580}/A_{580+\text{CHP}}\right)*1\;\mu l\;{\text{CHP}}_{1\;\text{nmol}}*200\;/\;V*5$$

in which 200 is the total sample volume, *V* is the added sample supernatant volume (15 μl), and 2 is the dilution factor with methanol (1:5).

#### 2.6.1 Proteotoxicity

As a proxy for proteotoxicity, the level of the stress protein 70 family (Hsp70) relative to the control was used. Frozen snails were homogenized individually on ice in extraction buffer (80 mM potassium acetate, 5 mM magnesium acetate, 20 mM HEPES, and 2% protease inhibitor at pH 7.5) according to their body mass (2 μl buffer/mg snail) and centrifuged for 10 min at 20,000 g and 4°C. To determine the total protein content of each sample, the protein‐dye binding assay of Bradford (1976) was used. Constant protein weights (40 μg per sample) were separated by minigel SDS‐PAGE (12% acrylamide, 0.12% bisacrylamide, 30´ at 80 V and 75′ at 120 V) and transferred to nitrocellulose membranes by semi‐dry blotting. The membranes were blocked in a 1:2 mixture of horse serum and TBS (50 mM Tris pH 5.7, 150 mM NaCl) for 2 hr. Subsequently, the membranes were incubated in the first antibody solution containing a monoclonal α‐Hsp70 antibody (mouse anti‐human Hsp70, Dianova, Hamburg, Germany; dilution 1:5,000 in 10% horse serum in TBS) on a laboratory shaker at room temperature overnight. Membranes were washed for 5 min in TBS and subsequently incubated in the second antibody solution (goat anti‐mouse IgG conjugated to peroxidase; Jackson ImmunoResearch; dilution 1:1,000 in 10% horse serum/TBS) on a laboratory shaker for 2 hr at room temperature. Following another washing step in TBS, the developed antibody complex was detected by staining with a solution of 1 mM 4‐chloro(1)naphthol, 0.015% H_2_O_2_, 30 mmol/L Tris pH 8.5, and 6% methanol. The optical volume [area of the bands (number of pixels) × average grayscale value after background subtraction] of the Western blot protein bands was quantified using a densitometric image analysis system (Image Studio Lite, LI‐COR Biosciences). For each sample, data were related to an internal Hsp70 standard (extracted from a pool of additional *T. pisana* unmanipulated individuals) to ensure comparability.

### 2.7 Morph frequencies and shell pigmentation intensity in 35 Mediterranean sites

To compare the morph communities in large populations of *T. pisana* to the population at the experimental site at Montfavet, a total of 35 Mediterranean sites with *T. pisana* populations (including the one at Montfavet) were randomly selected, covering the Mediterranean from west to east. In view of realistic global warming simulations for the next decades, we limited our selection to sites with an average temperature of the warmest quarter of the year that was maximum 2.7°C warmer than the Montfavet site (Table 1). At these sites, an average of 114 (36–237) living individuals were randomly sampled in summer and autumn 2018, and the frequency of morphs and the average pigmentation intensity in the population were recorded.

**Table 1:** Sampling sites, their geographical location, and data on the pigmentation of abundant *T. pisana*

Country	Province	Site	GPS latitude	GPS longitude	Δ*T* [°C] versus Mf	*n*	Whites %	Average pigmentation ± *SD*
Greece	Kriti	Agios Ioannis Knossou	35.3176	25.1518	1.87	139	97.14	188.03 ± 13.03
Greece	Kriti	Arkalohori	35.1573	25.2426	0.80	127	99.22	190.55 ± 11.16
Greece	Kriti	Keratokambos	34.9989	25.3747	2.00	121	95.00	194.58 ± 13.92
Greece	Kriti	Arvi	34.9911	25.4451	2.62	115	93.10	200.73 ± 13.67
Greece	Kriti	Matalla	34.9957	24.7549	2.50	141	96.18	181.59 ± 13.54
Greece	Makedonias‐Thrakis	Thessaloniki Angelohori	40.4945	22.8224	1.42	113	93.50	174.27 ± 13.77
Greece	Makedonias‐Thrakis	Thessaloniki Airport	40.5180	22.9932	1.43	115	98.41	172.01 ± 14.61
Greece	Makedonias‐Thrakis	Thessaloniki Zeda	40.5551	22.9801	1.42	135	94.59	163.55 ± 14.97
Greece	Makedonias‐Thrakis	Thessaloniki Krini	40.5767	22.9446	1.25	93	99.08	177.91 ± 13.03
Greece	Makedonias‐Thrakis	Thessaloniki—University	40.6351	22.9556	1.40	108	100.00	159.13 ± 13.93
Greece	Thessalía	Volos—Pefkakion	39.3463	22.9417	1.87	99	87.88	181.00 ± 9.78
Greece	Thessalía	Volos—Leman	39.3157	22.9286	1.88	101	84.16	173.75 ± 12.98
Greece	Thessalía	Volos—Nees Pagases	39.3275	22.9273	2.03	155	84.97	179.98 ± 13.58
Greece	Thessalía	Volos—Athinon	39.3382	22.9383	1.87	144	83.92	178.72 ± 11.19
Greece	Thessalía	Volos—Mall	39.3561	22.9276	2.05	126	94.44	174.15 ± 14.04
France	Prov.‐Alpes‐C. d´Azur	Montfavet	43.9159	4.8960	0.00	99	79.42	162.25 ± 18.23
Italy	Calabria	Crotone	39.1912	17.1386	1.67	188	18.78	152.31 ± 22.40
Italy	Toscana	Marina di Grosseto	42.7378	10.9512	0.08	36	2.78	110.06 ± 13.70
Italy	Toscana	Tombolo della Giannella	42.4678	11.1859	0.23	87	35.23	114.23 ± 17.24
Italy	Toscana	Tombolo di Feniglia	42.4082	11.2087	0.15	70	31.25	127.42 ± 21.52
Italy	Toscana	Fosso di Bolgheri	43.1914	10.5369	0.00	53	18.87	149.92 ± 18.35
Italy	Toscana	Bibbona	43.2379	10.5280	0.15	58	10.34	128.93 ± 19.24
Italy	Toscana	Castagneto Carducci	43.1795	10.5383	0.08	55	3.64	116.49 ± 16.04
Spain	Granada	Atarfe	37.2181	−3.6834	1.20	80	77.91	137.32 ± 18.17
Spain	Huesca	Ballobar—Alcanadre	41.6205	0.1948	0.68	123	97.54	153.98 ± 11.65
Spain	Huesca	Ballobar—Cinca	41.6135	0.2121	0.62	159	87.34	164.64 ± 12.65
Spain	Menorca	Lloc de Monges	39.9547	3.8491	0.73	103	46.60	126.75 ± 18.08
Spain	Menorca	Castell de Sant Nicolau	39.9956	3.8267	0.70	102	60.78	141.00 ± 18.74
Spain	Menorca	Santa Rita	40.0080	3.8457	0.72	106	16.98	114.72 ± 18.39
Spain	Menorca	Mahón	39.8870	4.2430	0.77	106	76.64	141.22 ± 21.61
Spain	Menorca	Montefí	40.0044	3.8602	0.67	100	44.00	122.48 ± 12.41
Spain	Menorca	Binixica	39.8722	4.1866	0.57	152	80.26	136.28 ± 17.08
Spain	Zaragoza	Alagon—La Ciudad	41.7682	−1.1126	0.11	237	82.35	145.86 ± 14.86
Spain	Zaragoza	Pinseque—La Ciudad	41.7430	−1.0970	0.10	126	88.19	164.92 ± 15.86
Spain	Zaragoza	Pinseque—El Calvario	41.7415	−1.1120	0.20	127	78.29	158.71 ± 16.15

Note

Δ*T* versus Mf: difference in the mean annual temperature versus the test site at Montfavet; *n*: number of sampled individuals; whites: percentage of unpigmented individuals in the population; average pigmentation of the shell on a scale between 0 (black) and 255 (white).

To determine morph frequencies, the “naturally white” (W) and the “naturally pigmented” (P) morphs were separated and counted for every population. Pigmentation analysis, which provides more detailed information on the shell's pigmentation intensity, was conducted as follows. Animals were killed in 70% ethanol, the shell cleaned from soil and plant matter, and indirectly illuminated in a standardized way on a photographic reproduction apparatus by four illumination sources with a natural light spectrum, avoiding any shadow. Photographs of both sides (apical and umbilical) of every shell were taken and converted into b/w by GIMP. Every shell picture was manually circumscribed on a graphical pad (the shell opening was excluded from analysis), and these image sections analyzed planimetrically and densitometrically (after background correction) using ImageJ 1.52a on Java 1.8.0. For every individual, the pigmentation of the shell was calculated considering the different shell areas and colorations of both sides. Pigmentation is given on a scale from 0 (black) to 255 (white).

To extrapolate from the current morph‐specific mortality at the Montfavet field site to future morph composition under the conditions simulated in the OTC experiments (temperature increase by 1.12°C), the surplus mortality rate in the naturally pigmented morph compared with the white morph was calculated for the current field conditions at Montfavet (f_field_) and the OTCs (f_OTC_). The factor by which the mortality of naturally pigmented individuals will increase at + 1.12°C warming at Montfavet was calculated by,

$$f_{\text{warming}}=f_{\text{OTC}}/f_{\text{field}}$$

This factor was subsequently used to calculate the future morph frequencies and the average pigmentation intensity of the *T. pisana* population at Montfavet, assuming a 1.12°C warming.

## 3 RESULTS

In summary, climate data revealed that the magnitude of global warming was simulated realistically in the OTCs. All biological data obtained for the different morphs showed that gradually more intense pigmentation poses increasingly strong physiological disadvantages under the tested conditions in the Mediterranean climate, particularly under conditions of simulated warming.

### 3.1 Climate data, exposure conditions, and snail characteristics

**Figure 2:**
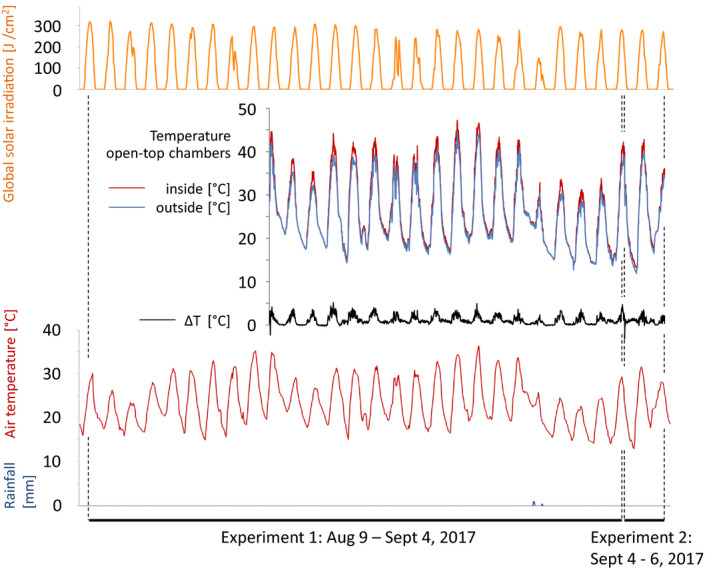
Climate data for the experimental site (Montfavet, Southern France, 43.916857 N, 4.877207E) during the time of the global warming simulation experiments (9 August–6 September 2017). Top graph: global solar irradiation [J/cm^2^] (orange). Middle graph: temperature [°C] at site 5 cm above ground: inside open‐top chambers (simulation of global warming, red) and outside open‐top chambers (blue). Mean *T* outside the chambers during daytime (9–21 hr): 30.98 ± 5.76°C, max *T*: 44.50°C, and mean *T* outside the chambers at night (21–9 hr): 19.52 ± 3.19°C. Mean *T* inside chambers during daytime: 32.68 ± 6.39°C, max *T*: 47.20°C, and mean *T* inside chambers during the night: 20.06 ± 3.21°C. The delta between inside and outside the chambers is depicted in black: mean Δ*T*: 1.12 ± 0.94°C and max Δ*T*: 5.20°C. Bottom graph: air temperature [°C] in the shadow at 1 m above ground as recorded in Avignon climate station 50 m away from the experimental site (red), precipitation [mm] at this site (blue)

Climate data during exposure in the OTCs are displayed in Figure 2. The experimental period was dominated by constantly sunny days with almost zero precipitation. The mean temperature inside the open‐top chambers (OTC) at 5 cm above ground level was on average 1.12°C higher than outside. During the day (09–21 hr), the difference was +1.69°C, and during the night (21–09 hr), it was +0.54°C. The maximum difference was +5.20°C, and the maximum temperature inside the OTC was 47.20°C. Linear regression analysis of the long‐term climate data obtained from Avignon station (30 years from January 1988–December 2017) revealed an average increase in the monthly maximum of +0.0033658°C/month resulting in +1.21°C within these 30 years. In parallel, the monthly means increased by +0.0037265°C/month to a warming of +1.34°C within 30 years. Therefore, the magnitude of temperature differences in our experiment can be regarded a realistic simulation of global warming.

**Figure 3:**
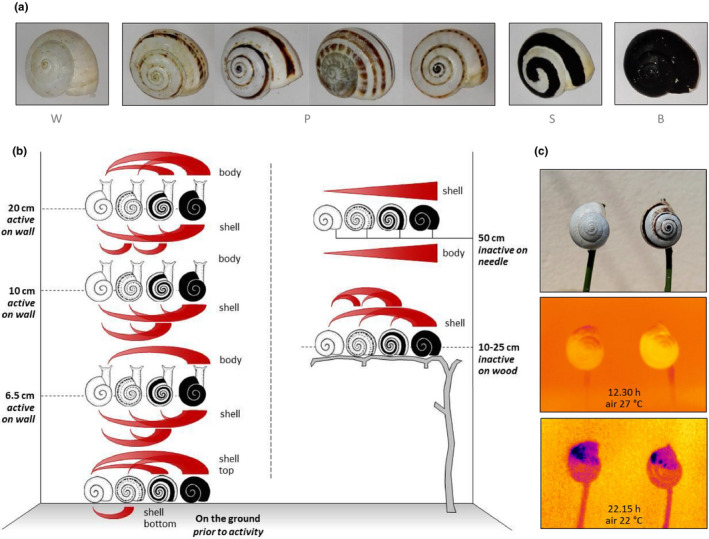
*Theba pisana* morphs and corresponding internal/shell temperatures in different situations in the field or the global warming experiment. a: Representative individuals of the morphs “naturally white” (W), “naturally pigmented” (P), “artificially striped” (S), and “artificially blackened” (B). b: Significant differences in temperature of the four morphs exposed to different situations, either at the bottom or top of the shell, at the shell surface or at/inside of the soft body. Red wedges depict differences at *p* ≤ .05 with the wedges’ bigger part symbolizing the, respectively, higher temperature. Left panel: situation at different heights in open‐top chambers, on the ground or at different heights when moving up the chambers’ walls. Right panel: Situation in an inactive state on vertically arranged wooden sticks inside the open‐top chambers or at 50 cm above ground on a needle thermometer in the field. Curved wedges are based on significances obtained by pairwise comparisons, and straight wedges are based on ANOVAs. When lying on hot ground thermal conduction heated up the shell (bottom) independent of its coloration. When snails flee, the heat by moving upward the chambers’ walls darker shells tend to become warmer than white ones, which also correspond to the respective body surface temperatures when the snails reach positions more distant to the soil. This phenomenon was corroborated by data obtained for individuals, which deliberately climbed wooden sticks inside the open‐top chambers or which were measured under field situations at a constant 50 cm distance from soil. c: Two individuals of morphs W and P of the same size and origin in direct vicinity to one another at identical distance from the soil (top), and thermographic images when exposed to solar radiation around noon (12.30 hr at 27°C air temperature, middle) or in the evening (22.15 hr at 22°C, bottom). The shell surface of the pigmented morph displayed higher temperatures at all parts, compared with the white one, both in the middle of the day and in the evening. As well, in both morphs, the side directed to the ground was warmer than the top side indicating the importance of indirect thermal radiation reflected by the ground. Increasing temperatures are depicted in the order blue < purple < orange < dark yellow < bright yellow

The initial weight of the snails introduced into the OTC did not differ among the four morphs. None of the multicomparisons were significant (Tukey‐corrected). In contrast, the pigmentation intensity of all four morphs (Figure 3a) differed significantly at *p* < .0001 (Bonferroni‐corrected; W: 223.32 ± 9.82, P: 199.04 ± 18.46, S: 156.89 ± 9.34, B: 58.11 ± 10.79).

### 3.2 Warming of snails

Data on the warming of snails obtained in all three experiments (escape behavior, OTCs, and exposure experiments at 50 cm above the ground) all showed that darker shells heat up more than paler ones.

In the escape behavior experiments, darker shells became significantly warmer than paler ones, prior to moving and on their way upwards (Figure 3b, left panel). Even in contact with the hot ground, at least parts of the shell of all (naturally and artificially) pigmented morphs became significantly warmer than those of the white ones. When moving upwards, shells of darker snails reached higher temperatures at all heights. At 20 cm above ground level (occasionally also at 6.5 cm), the soft body of artificially painted morphs (S, B) was also warmer than that of natural ones (W, P).

In addition, thermography of snails that escaped from the soil surface in OTCs at identical conditions revealed significant differences in external shell temperatures between the morphs. Shell temperature decreased in the order W < P < S < B (all significant, Welch's test, Bonferroni‐corrected; Figure 3b, right panel). Because shell temperatures of climbers were higher on the side directed to the ground (Figure 3c), and thus most likely depend on thermal emission from the hot soil, we also assessed shell temperature of the different morphs in relation to the maximum soil temperature in each OTC. We found that shell temperatures decreased in the order W < (P = S) < B (all significant except for P vs. S, Welch's test, Bonferroni‐corrected, data not shown).

These results were confirmed by thermography and needle thermometer measurements at 50 cm above ground level (Figure 3b, right panel). With increasing difference in the pigmentation of two neighboring snails, the temperature difference increased accordingly. This held true for the external temperature of the shell (*p* = .0211, *F* = 10.9851, max. effect size 2.73°C [W vs. B]) and for the internal temperature in the soft body (*p* = .0486, *F* = 6.7265, max. effect size 1.92°C [W vs. B] both linear regression, ANOVA).

### 3.3 Behavior

The escape behavior experiments with field‐collected, non‐OTC‐exposed animals showed that the ability of snails to escape from a hot soil surface did not differ consistently among the four morphs. The rank sum analyzes for the parameters “start to move,” “start to climb,” and “reaching 20 cm in height” revealed just a slight but insignificant trend to darker individuals moving (*p* = .1778, *F* = 2.4570, linear regression, ANOVA), climbing (*p* = .1940, *F* = 2.2484), and reaching 20 cm in height (*p* = .2538, *F* = 1.6618) earlier than paler ones (data not shown).

Within the OTC experiment, where snails had the opportunity to climb skewers and escape the surface heat, behavior of the snails rarely altered as a result of morph coloration. Numbers of snails taking positions “>10 cm” only differed in later time points (Poisson GLMM *p* < .05), with B < (P and W) from day 5, B < S from day 7, and S < (P and W) from day 9. However, numbers did not vary significantly (*p* > .05) with morph color when a binomial model was used, an analysis that took into account the number of individuals alive in the OTCs, indicating that morph‐specific basic mortality was crucially impacting on the total number of climbing snails.

For top positions reached between time points 2 and 5, in single‐competition OTC comparisons, we found two significant differences out of the possible six combinations (binomial GLMM *p* < .05). W morphs took up more top positions than B, and P took up more top positions than S. In terms of like‐for‐like “competitive response,” P took up more top positions than S. Also, comparing all individual combinations for effects of morph type, neighbor identity only played a significant role for S. Here, S took up more top positions when neighboring B individuals, but not when neighboring P or W. We did not find significant differences of overall competitive effect of morph coloration on the snails for either “>10 cm” or “top position occupancy.” Thus, morph color did not alter the climbing behavior of neighbors.

### 3.4 Stress physiology and biochemistry

**Figure 4:**
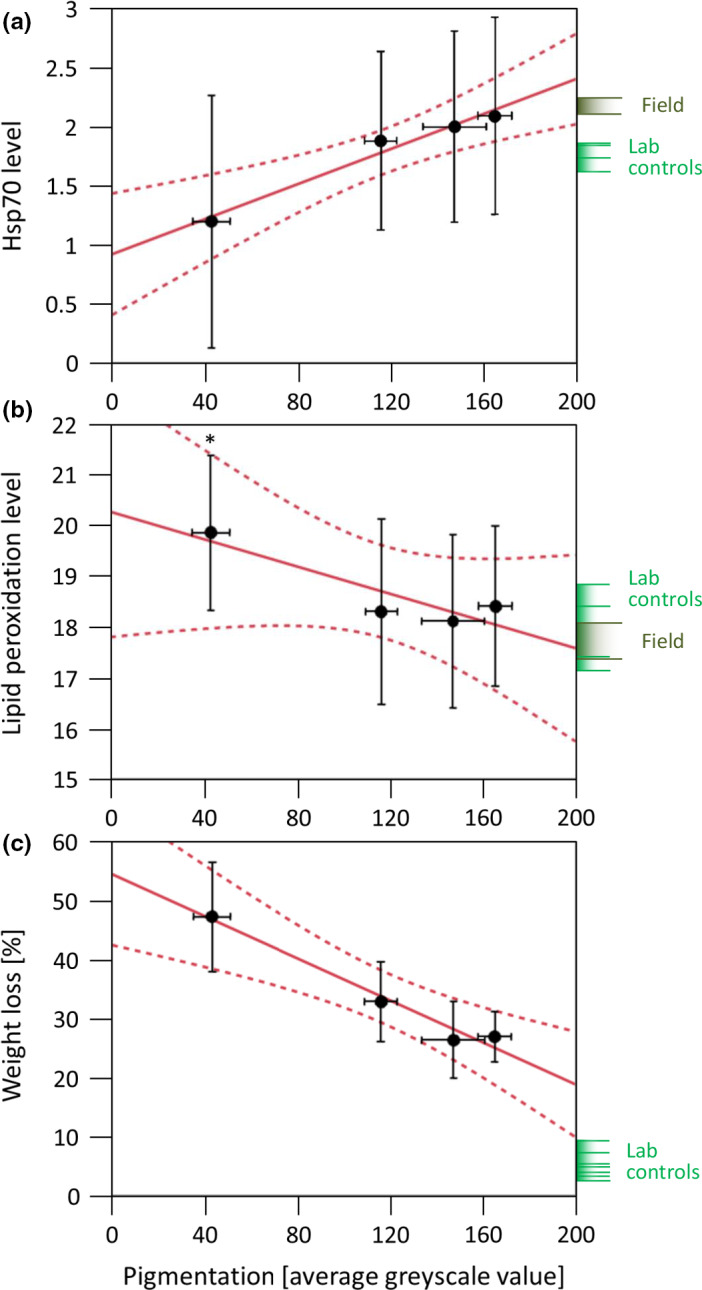
Levels of the protein‐stabilizing Hsp70 stress protein family (a: optical volume relative to standard) and of lipid peroxidation (b: cumol hydroperoxide (CHP) equivalents per mg wet wt., y‐axis truncated) after 3 days, and weight loss (c: % of initial wet weight) after 7 days of exposure to simulated global warming conditions in open‐top chambers in the two natural and the two artificial morphs of *Theba pisana* (means ± *SD*, respectively). Average shell pigmentation intensity (±*SD*) of the morphs was quantified on a grayscale from 0 [black] to 255 [white]. Data obtained for laboratory controls (without exposure to direct sun at a constant 26°C) or field specimens in their natural environment at Montfavet are displayed at the right margin of the graphs (each bar represents a sample, and shaded area indicates the range between min and max). Linear regression analysis and 95% confidence intervals. Data showed a significant decrease in the chaperoning system (ANOVA, *p* = .0158) and a significant increase in weight (presumably water) loss (ANOVA, *p* = .0149) with increasing shell pigmentation. *The lipid peroxidation level in artificially blackened individuals was significantly increased (0.0001 ≤ *p *≤ .0039 vs. the other morphs in the open‐top chambers and *p* < .0001 vs. the laboratory control)

After a 3‐day exposure in the OTCs, the Hsp70 level of the snails was significantly linearly correlated with the shell pigmentation intensity of the morphs and Hsp70 content (Figure 4a). The more intense the shell pigmentation, the higher was the destruction of the capacity of the Hsp70 system (linear regression, ANOVA). This correlation held true for datasets either including moribund individuals with zero Hsp70 levels (*p* < .0001, *F* = 40.1127) or excluding them (*p* = .0003, *F* = 13.2879). The Hsp70 level of laboratory controls and field‐collected individuals did not differ much from OTC‐exposed morphs W, P, and S, but B morphs showed a comparatively (though insignificantly) lower level of stress protein.

Concomitant with the breakdown of the Hsp70 chaperoning system, black morphs showed significantly increased lipid peroxidation after 3 days in the OTCs (Figure 4b). This effect was evident with respect to the other OTC‐exposed morphs (B > [W = P = S], *p* between .0001 and .0039) and vs. blacks of the laboratory control (*p* < .0001, *F* = 5.6172, ANOVA and Tukey–Kramer). Also for this parameter, the laboratory controls and field‐collected individuals did not differ much from OTC‐exposed morphs W, P, and S, but B morphs showed a significantly elevated lipid peroxidation level (*p* < .0001, *F* = 5.6172, ANOVA and Tukey–Kramer).

In the OTC experiment, morphs differed significantly with respect to their weight loss at day 8 (Figure 4c). ANOVA/Tukey–Kramer analysis showed S and B morphs to lose more weight than W and P. Black morphs lost significantly more weight than S morphs ([W = P] < S < B, p between <.0001 and .0113, *F* = 46.7770, Figure 4). In the mixed models, all pairwise comparisons involving black‐shelled snails were highly significant, that is, B individuals on day 8 weighed far less than snails of any other color (*p* < .001 for all comparisons). Pairwise comparisons between other shell colors were nonsignificant (P vs. S: *p* = .344; W vs. P: *p* = .977; W vs. S: *p* = .535). Generally, all OTC‐exposed snails had lost significantly more weight than the laboratory controls (*p* < .0001, *F* = 46.7770, ANOVA and Tukey–Kramer).

### 3.5 Mortality

**Figure 5:**
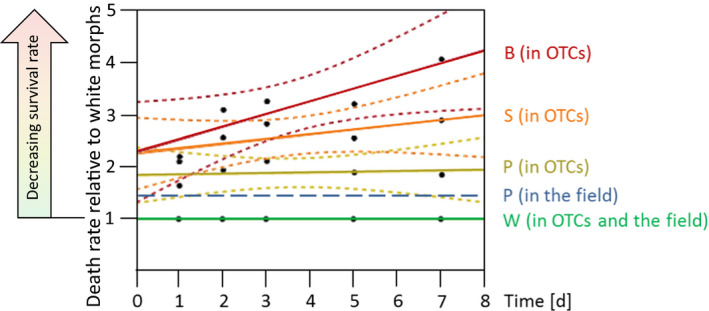
Mortality of naturally and artificially pigmented *T. pisana* morphs in the field population and the global warming‐simulating experiment at Montfavet, Southern France, relative to the white morph (green, set to 1.0). The mortality of naturally pigmented (P) individuals in the natural situation is 148% of that of whites (blue dashed linear regression line). In case of experimental warming in open‐top chambers of 1.12°C on the average, the mortality rate of P morphs increases by a factor of 1.26%–187% of the whites, on the average (yellow‐green, linear regression with 95% confidence intervals). The mortality rates in artificially striped (S, orange line) and blackened (B, red line) individuals increased with time of exposure to simulated global warming and showed that these morphs would not persist under these conditions

In the OTCs, the four morphs differed a lot in rates of mortality (Figure 5). Already at days 3 and 5, the survival rate of white morphs was significantly higher than that of all other morphs (*p* ≤ .001, *F*_day 3_ = 19.8396, *F*_day 5_ = 24.0851, max. factor 1.37 (W vs. B), ANOVA and Tukey–Kramer). In addition, at the same time, the mortality was higher in B than in the naturally pigmented morph P (*p* ≥ .0076, *F*_day 3_ = 19.8396, *F*_day 5_ = 24.0851, max. factor 1.71, ANOVA and Tukey–Kramer). Regression analysis revealed increasing mortality in both artificially colored morphs B and S relative to whites with proceeding time, starting right at the beginning of exposure in the OTCs. Mortality in naturally pigmented P individuals was also higher than in W when exposed to the OTCs for a week (*f*_OTC_: on the average 1.87 times the W mortality with a very slight incline, Figure 5). At the field site Montfavet, the mortality of individuals, indicated by the percentage of dead snails detached from plants (not on the soil surface), was higher in the P morph (5.93% dead snails on plants, *n* = 540) than in W (4.01%, *n* = 1,620), leading to a factor f_field_ of 1.48 times the mortality in P versus W under current climate conditions in 2017.

The time to 50% mortality was significantly different in all multicomparisons for the competitive response of individuals of morph colors (gamma GLMM *p* < .0001 to *p* = .012). In like‐for‐like comparisons of black versus white competitive responses, black individuals reached 50% mortality in only 4.91 days, compared with 10.08 days for white individuals. Overall, black individuals died quickest, subsequently followed by S (which was similar to B but significantly different), P (very different to S by 2 days), and W (2.5 days more than P).

Mixed models also revealed significant differences in the lethal competitive “response” of morph over the first nine days in the OTCs (“mortality rate,” binomial GLMMs): The slopes of the models showed that B snails died quickest, followed by S (which was significantly different to B: *z* = −5.58; *p* < .0001), then W and P were the slowest, both significantly slower than S (W vs. S *z* = −4.422, *p* < .0001; P vs. S *z* = −4.276, *p* < .0001). In the mixed models, the death rate response of the P morph was a little quicker (like in the regression analyzes) but not significantly different from W (*p* = .269).

In terms of competitive effects, snail color hardly played a role in the mortality of neighboring snails. Most pairwise comparisons were nonsignificant. There were no significant differences for time to 50% mortality, and only one significant result for mortality rate (B had more effect than P, *p* = .0315). Therefore, the impact on a neighbors’ mortality was generally independent of morph pigmentation.

In respect to the relevance of size for survival, we did not find early mortality of particularly small or large individuals in any morph (Welch's test, Bonferroni‐corrected).

### 3.6 Morph frequencies and pigmentation intensity in Mediterranean populations

**Figure 6:**
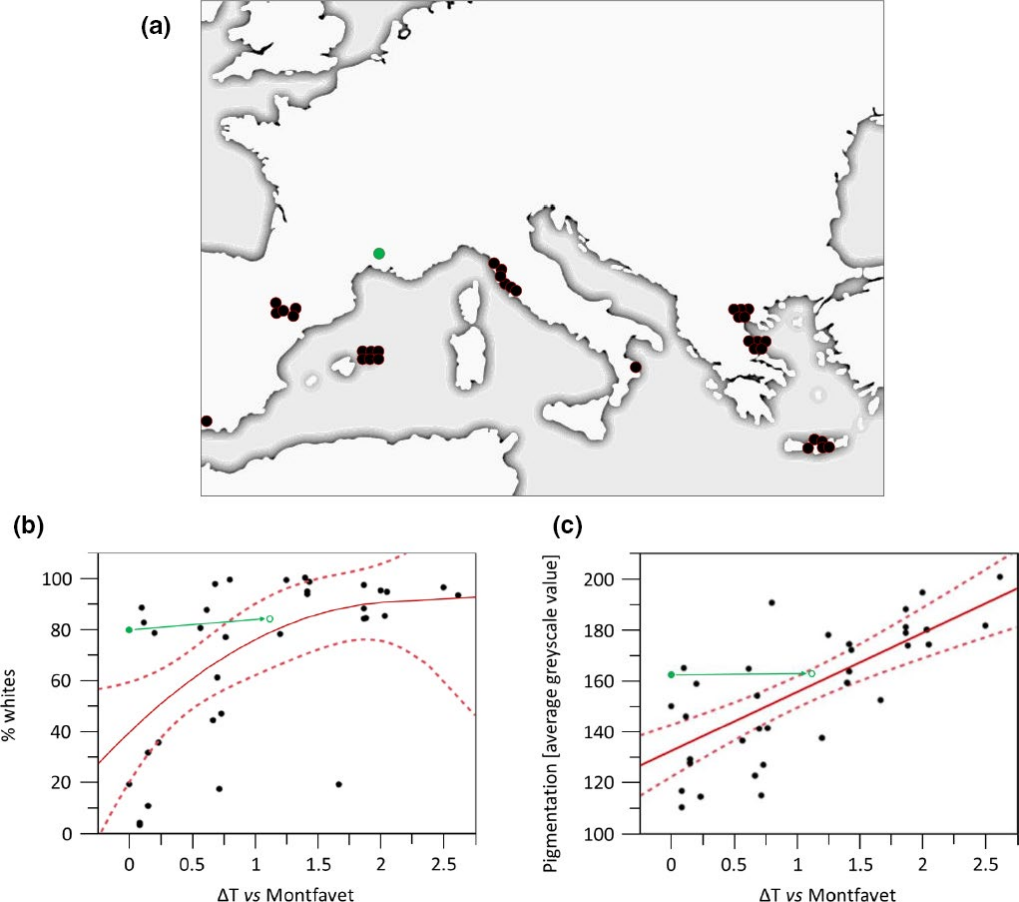
Field locations, morph frequency, and pigmentation intensity in abundant *T. pisana* populations. (a) Locations of the test site in Montfavet (green) and of 34 other Mediterranean locations that host *T. pisana* populations and are already up to 2.7°C warmer than Montfavet in respect to the average temperature of the hottest quarter of the year (black‐red). (b) Current percentages of white individuals in the *T. pisana* population at Montfavet (green dot) and the other 34 Mediterranean locations (black dots) versus the mean ΔT the hottest quarter of the year is warmer than Montfavet. (c) Average shell pigmentation intensity in the *T. pisana* population at Montfavet (green dot) and the other 34 Mediterranean locations (black dots) versus the mean Δ*T* the hottest quarter of the year is warmer than Montfavet. Temperature data for (b) and (c) were obtained from WorldClim.org. Nonlinear or linear regression curves and 95% confidence intervals (red) display a strong trend toward a dominance of paler/white individuals at sites with increasing summer temperatures. The green circle displays the shift of the population at Montfavet as calculated from the mortality results obtained in this study for the simulated warming of 1.12°C. The rather exceptional current position of the Montfavet data point (which will shift to the average trend with ongoing global change) is in accordance with the exceptionally high degree of thermal pressure change this population has been facing during the last 30 years: +1.34°C, in comparison with an average of +1.1°C in the entire Mediterranean

In the 35 investigated *T. pisana* populations (sampling locations shown in Figure 6a), morph frequencies and average pigmentation intensity varied considerably, with frequencies of whites between 2.78% and 100%, and pigmentation intensity values between 110.06 and 200.73 (Table 1). Both parameters showed a significant correlation with the mean temperature of the hottest quarter of the year (Figure 6b,c). The frequency of the white morph increased with increasing Δ*T* and followed a saturation curve approaching almost 100% whites (*p* = .0015, *W* = 7.9873, ANOVA). In addition, *T. pisana* from sites warmer than Montfavet were found to be on average paler with increasing Δ*T* (*p* < .0001, *W* = 35.1335, ANOVA).

Based on the observed mortality rates in the two natural morphs, W and P, in the OTCs and the field site at Montfavet, we extrapolated the morph frequencies and the mean pigmentation intensity at this site assuming an additional temperature increase of 1.12°C. According to the calculated values for *f*_OTC_ and *f*_field,_ the average mortality of the P morph (vs. W) will further increase by the factor of 1.264 (*f*_warming_). Therefore, an increase in the average temperature by 1.12°C will result in a shift in the frequency of naturally pigmented individuals from today's 20.58 ± 2.74% to 16.28 ± 2.17% and for the white morph from 79.42 ± 2.74% to 83.72 ± 2.17%. Thus, with 1.12°C warming we predict a loss of 20.9% of pigmented individuals at this site and a slight increase in the average pigmentation value by 0.48% toward “white” (arrows in Figure 6b,c).

## 4 DISCUSSION

The role of shell pigmentation in the physiology and evolution of land snails is among the best‐studied and most discussed topics in animal evolutionary ecology. Whereas numerous publications have addressed aspects of the impact of thermal stress, they mainly focused on hν absorption and heat transfer or on the distribution of morphs in particular habitats (reviewed by Schweizer et al., 2019). In our current study, we highlight three innovative aspects that have not been previously addressed:

- The joint analysis of multiple parameters, collated in a syndrome across different levels of biological organization (thermodynamics, biochemistry, physiology, behavior, survival rates, and the population frequencies of shell banding morphs), allowing us to trace thermal effects from the molecular to the population level.
- An experimental approach to mechanistically show the effects of environmental warming on land snails, which has not been conducted before in such complexity.
- The extrapolation of our results to predict the consequences of environmental warming for future morph frequencies in a field population in the Mediterranean.

Our results, obtained in experiments tailored to suit our specific research questions, consistently suggest that *T. pisana* individuals with pigmented shells, in both the current Mediterranean climate and under simulated environmental warming, become warmer than white ones, thus experiencing additional thermal stress. This warming effect was most pronounced in the artificially painted individuals, but even the shell and body of the naturally pigmented morph were significantly warmer than those of nonpigmented individuals. Previous studies investigating heat stress on *Theba*, *Cepaea*, and *Littoraria* (Cook & Freeman, 1986; Hazel & Johnson, 1990; Heath, 1975; Jones, 1973; Knigge et al., 2017) generally compared fully banded with unbanded morphs. Our results not only confirm the findings from those studies, but also elaborate on them to include strong experimental manipulations of color that define the mechanism of the warming. Another recent study has found the same trend for faintly pigmented *T. pisana* individuals versus white ones, even under moderate illumination in the laboratory (Tull et al., 2020). At least under Mediterranean climate conditions, at present and in the future, there is no doubt that dark individuals will heat up more intensely than pale ones.

Evidence is thus mounting that differently pigmented morphs experience different shell and body temperature in a given situation: Darker morphs warm up more quickly and more intensely. It has long been known that snails respond to elevated body temperature by selecting preferred substrate temperatures (Chapperon & Seuront, 2011; Riddle, 1990), or by climbing tall objects to escape from substrates with extreme temperatures (Cowie, 1985; Di Lellis et al., 2012; McQuaid et al., 1979). These responses may to a certain degree buffer the physiological impacts of heat (Ng et al., 2017). Climbing objects often results in aggregates of multiple individuals. Depending on the climate, the aggregate centers are either warmer (in cold climate, Chapperon & Seuront, 2012) or cooler (in hot climate, McQuaid et al., 1979) than the aggregate surfaces. An alteration in temperature due to climbing or within aggregates implies that there may be intraspecific competition to find the “best place” for temperature regulation. However, Chapperon and Seuront (2012) reported that all snails of a cluster benefit from the aggregate. The question thus arises whether either fighting for an optimal resting place or joint action (i.e., “co‐operation”) between individuals has been evolutionary favored, or whether any trade‐offs in other traits exist. Particularly in the Mediterranean region, differently colored snails may need to possess different abilities to escape the soil surface. However, we did not find any morph‐specific behavior. While darker morphs heated up more quickly, they did not flee the hot surface any quicker than pale morphs, nor did they climb more quickly, either during observed “racing” experiments, or as determined from the number of top positions occupied on the skewers in the OTCs. Yet, by analyzing the earlier time points—which takes into account mortality—we found minor evidence in the opposite direction to the “fleeing heat” hypothesis. In general, while very few results were significant, the evidence suggested that paler individuals generally reached higher positions on the skewers than darker individuals, but with little effect on overall mortality. There was even less evidence to suggest that morph coloration had any consistent competitive effect (in terms of neither climbing nor mortality). Based on our results, we thus conclude that competition among morphs is at most indirect and may just be a consequence of limited space, supporting no particular morph.

Among the main biochemical effects of elevated body temperature is the impaired integrity of intracellular proteins and cell membranes. As protein integrity is mandatory for a cell's survival, a biochemical chaperoning machinery that assists in protein (re)folding has been established early in the evolution of life. A central component of this machinery is the Hsp70 protein family, whose induction by heat and drought has been well characterized in land snails (Arad et al., 2010; Di Lellis et al., 2014; Dieterich et al., 2013, 2015; Gaitán‐Espitia et al., 2013; Köhler et al., 2009; Kotsakiozi et al., 2015; Mizrahi et al., 2010, 2011, 2012a, 2012b, 2015, 2016; Scheil et al., 2011; Troschinski, et al., 2014). As long as an induction of Hsp70 may compensate for the proteotoxic impact of body temperature elevation, organisms can limit pathologies and ensure survival. A breakdown of the Hsp70‐based refolding machinery reflected by declining levels of this chaperone, however, is indicative for oxygen limitations in the body fluids at high temperatures, resulting in oxidative stress (Pörtner, 2002) and subsequent destructive processes in tissues that, on the long run, lead to elevated morbidity (Eckwert et al., 1997). In our OTC experiment, we found that simulated environmental warming gradually decreased the Hsp70 level in morphs with increasing pigmentation intensity, symbolizing a collapsing chaperoning system, particularly in the blackened snails but also, tentatively, in the other morphs. Concomitant with this temperature effect on proteins, the lipid peroxidation level increased with increasing pigmentation intensity of the morphs as a consequence of elevated respiration and thus oxygen consumption in snails with elevated body temperature (Pörtner, 2002). The disruption of redox homeostasis, however, generally is a key phenotype of pathological conditions in many organisms and cell types, as excessive lipid peroxidation alters the physical properties of cellular membranes and can result in covalent modification of proteins and nucleic acids (Gaschler & Stockwell, 2017). The association between high temperatures and lipid peroxidation in land snails has experimentally been shown before in *Xeropicta derbentina* individuals that have been exposed to 38–48°C for 8 hr (Dieterich et al., 2015; Troschinski, et al., 2014), but it is noteworthy that, in our study, even a rather moderate increase in elevated temperatures induced lipid peroxidation in a morph‐specific manner.

In addition to the biochemical stress posed by elevated body temperature, all animals exposed to simulated environmental warming lost more weight than the controls. Weight loss in this context is almost exclusively due to the loss of water, and the impact of catabolic processes is neglectable within a rather short time frame (Reuner et al., 2008). Particularly, the artificially colored morphs B and S suffered from water loss >30% (wet wt.) within a few days, confirming the results of Moreno‐Rueda (2008) who has painted *Sphincterochila candidissima* black. Highly effective water retention, however, seems to be essential for land snails living in arid and semi‐arid environments, which makes for instance the desert snail *Sphincterochila boissieri* to lose only 0.45 mg water per day, allowing survival of up to 4 years in the drought (Schmidt‐Nielsen et al., 1971). Kotsakiozi et al. (2015) showed that only one of six *Codringtonia* species lose water during summer estivation, and *Cantareus apertus*, which estivated for 6 months had only 14% less water than active individuals (Reuner et al., 2008). Plausibly, the higher internal temperature in the intensely (and artificially) colored morphs of our study caused a higher need for evaporative cooling, which considerably decreased the water content of the snails' soft bodies.

The syndrome of thermal stress, which comprises higher internal temperature, weakening of the protein chaperoning system, enhanced peroxidation of membranes, and increased loss of water in the pigmented morphs, feasibly resulted in differences in the survival rate among morphs. This was particularly obvious under conditions of environmental warming in the OTCs, but was already visible in the field. Even though, in our experiments, inactivity of climbed snails was disturbed once a day for a few minutes—an additional stress factor which is potentially relevant also in the natural habitat—the relative difference in water loss and mortality among the morphs was striking: The OTC experiments revealed that, in a situation that simulated the global warming effect, the brighter the shell of an individual the higher is the chance for its survival. This was independent of its size or neighboring morph, provided that there was enough space to climb up and escape the hot soil. Thus, the capacity of a morph to occupy top positions on a vertical object did not determine survival but was rather a consequence of it. The experimental design using artificial painting helped to reveal the general association between darker pigmentation and higher mortality, but this effect was not restricted to the artificially colored snails, but also visible in the naturally pigmented morph P, which had a 1.87 times higher mortality rate (and reached 50% mortality 2.5 days earlier) than the unpigmented morph W under simulated environmental warming conditions.

Even in Montfavet's climate of 2017, which was a snapshot in the ongoing process of global warming, the naturally pigmented morphs displayed a 1.48 times higher mortality than their white conspecifics. As *T. pisana* reproduces at the end of its lifetime in autumn and early dying individuals do not reproduce at all, such a difference in survival is of crucial relevance for the population structure. Hence, in their entirety, our results imply that the association between shell pigmentation intensity and thermal effects reflects causality for thermal selection pressure on pigmentation. To establish evidence for cause–consequence relationships between these parameters and across the different levels of biological organization (Triebskorn et al., 2003), we applied Hill's criteria of causation (Hill, 1965). The following criteria strengthened our interpretation.

- Strength of association: Pigmentation and a high number of thermal effects were significantly correlated at *p*‐values as low as <.0001.
- Coherence: Thermal effects all pointed toward the same direction: The darker the shell, the higher the internal and surface temperatures, the physiological challenge on protein and membrane integrity, the water supply, and the survival. For some of these parameters, also different methods of data analysis resulted in the same findings. Furthermore, results from OTC experiments and field observations were coherent.
- Consistency: Our findings are consistent with other studies reporting on increased thermal effects in dark phenotypes of snails (Cain & Currey, 1963; Cook & Freeman, 1986; Cowie, 1990; Cowie & Jones, 1985; Currey & Cain, 1968; Dieterich et al., 2013; Hazel & Johnson, 1990; Heath, 1975; Heller, 1981; Johnson, 1980, 2011, 2012; Jones, 1973; Jones et al., 1977; Moreno‐Rueda, 2008; Ożgo & Komorowska, 2009; Schilthuizen, 2013; Staikou, 1999).
- Specificity: Significant differences between the different morphs were only recorded in field and OTC studies with particularly high temperatures. There were no differences between the morphs in the laboratory controls, which had been kept at moderate temperatures.
- Plausibility: Connections between thermal effects at different levels of biological organization are thermodynamically, biochemically, and biologically plausible. Due to physical principles of light absorption, both the surface of darker shells and the interior of individuals with darker shells become significantly warmer than those of brighter ones. Elevated temperature is known to increase oxidative stress and protein degradation, thus elevated lipid peroxidation levels and the pronounced breakdown of the Hsp70‐based protein chaperoning system with increasing pigmentation can plausibly by explained. Furthermore, increasing body temperatures evidently demand intensified evaporative cooling efforts, thus result in a higher loss of body mass due to the loss of body water. In the next step of argumentation, unhampered membrane integrity, an intact intracellular stress response system counteracting protein breakdown, and sufficient water supply are prerequisites of survival of an individual. Therefore, impairment of these basic requirements for life could clearly explain elevated mortality rates in morphs with stronger pigmentation. Plausibility thus links thermodynamics, biochemical and physiological stress, and—via early mortality, which prevents reproduction—selection pressure on shell pigmentation intensity.

The consequences of this selection pressure are already reflected in the large field population at Montfavet that shows a higher mortality rate in naturally pigmented individuals than in white ones, even though the pigmentation intensity in the P morph was not very intense, compared with other Mediterranean populations.

Evolutionary aspects of thermal selection on shell coloration in land pulmonates have been addressed in the past, however, without investigating the thermodynamic, biochemical, and physiological fundamentals. There is consensus that snail populations in shaded habitats, less dominated by solar radiation, evolve toward darker shells than those in open habitats, and this effect has been reported for a number of species (Cain & Sheppard, 1954; Heller, 1981; Ożgo & Komorowska, 2009; Schilthuizen, 2013), despite remaining uncertainties about the (local) role of shell banding in, for example, thermoregulation (Cameron & Cook, 2012; Kerstes et al., 2019; Silvertown et al., 2011). The debate on the underlying mechanism for this phenomenon—thermal selection versus visual predation pressure by birds and rodents—is still ongoing (reviewed in Schweizer et al., 2019), but known geographic clines of particular phenotypes of land snails (Cowie, 1990; Jones et al., 1977) signify the relevance of climate parameters in this respect.

What will be the consequences for *T. pisana* and their different morphs at Montfavet and, eventually, the entire Mediterranean? It is highly unlikely that this species will go extinct in Southern France provided that environmental warming will be limited to about 2.5°C. This geographical area is not located at the edge of the environmental limits of *T. pisana*, and it has recently been shown (for 10,000 *Arabidopsis thaliana* genome‐wide SNPs) that the strongest climate‐driven selection is currently experienced by populations living at such edges (Exposito‐Alonso et al., 2019). Furthermore, in 136 case studies of possible climate change‐related extinctions, none of the proximate causes of global change have shown a relationship between local extinction and limited tolerance to high temperatures (Cahill et al., 2013), possibly because life history, behavior, and physiology can be altered by microevolution in response to environmental warming (Debecker & Stoks, 2019). The only proposed extinction of a snail, *Rhachistia aldabrae*, as a result of climate change had to be revised because this species was later rediscovered (Gerlach, 2007).

The variation in *T. pisana*'s shell pigmentation will probably persist in the future, either as a result of year‐to‐year change in the intensity of physiological pressure (Johnson, 2011, 2012) or as a result of buffering mechanisms within the limits of phenotypic plasticity (Köhler et al., 2013). However, we expect a decrease in the frequency of pigmented individuals in the Montfavet population with increasing temperatures. This prediction is based upon our results on the higher physiological pressure on the pigmented morph and the higher frequency of white individuals, concomitant with an average decrease in pigmentation intensity in 34 other *T. pisana* populations in the Mediterranean, which is related to increasing temperatures in the hottest quarter of the year. In our model, we solely used the selection against darker morphs to predict the future average pigmentation intensity of this population, even though phenotypic plasticity will most likely modify morphological variation also in a future population at this site. In this context, the broadness of heritable ranges of phenotypic plasticity in different genotypes will determine the number of genotypes that will go extinct in a warming environment and thus possibly limits the ability of the population to restore the full spectrum of phenotypic variation (Köhler et al., 2013). Quantitative information on the broadness of morphological reaction norms of given phenotypes, unfortunately, is completely lacking for *T. pisana*, which prevents inclusion of this aspect in a mathematical model. However, the close fit of the selection‐based prediction for the Montfavet population to the pigmentation data for already warmer sites indicates that the selection of phenotypes very probably outweighs phenotypic plasticity in a warming Mediterranean. Even in the colder climate of Wales, two thirds of the variation in the pigmentation pattern of *T. pisana*, documented by Cowie (1992), could be explained by the genetic basis and only about one third by environmental factors (Köhler et al., 2013).

We also propose that the selection against pigmented individuals at Montfavet has already been ongoing in the last years, because the pigmentation intensity in this population is comparably low and current differences in the morphs’ mortality rates are evident. In addition, the Avignon region has experienced exceptionally high warming during the last three centuries compared with the average in the Mediterranean, which already is a hot spot of global warming. Consequently, further temperature increases will probably not lead to the extinction of *T. pisana* in this area but will predictably reduce intraspecific diversity.

## CONFLICT OF INTEREST

None declared.

## AUTHOR CONTRIBUTIONS

**Heinz‐R. Köhler:** Conceptualization (equal); Data curation (equal); Investigation (equal); Writing‐original draft (lead). **Yvan Capowiez:** Conceptualization (equal); Data curation (equal); Investigation (equal); Writing‐review & editing (equal). **Christophe Mazzia:** Investigation (equal). **Helene Eckstein:** Investigation (equal). **Nils Kaczmarek:** Investigation (equal). **Mark Bilton:** Formal analysis (equal); Writing‐review & editing (equal). **Janne K. Y. Burmester:** Investigation (equal). **Line Capowiez:** Investigation (equal). **Luis J. Chueca:** Resources (equal). **Leonardo Favilli:** Resources (equal). **Josep Florit Gomila:** Resources (equal). **Giuseppe Manganelli:** Resources (equal). **Silvia Mazzuca:** Resources (equal). **Gregorio Moreno‐Rueda:** Resources (equal). **Katharina Peschke:** Formal analysis (equal). **Amalia Piro:** Resources (equal). **Josep Quintana Cardona:** Resources (equal). **Lilith Sawallich:** Investigation (equal). **Alexandra Staikou:** Resources (equal); Writing‐review & editing (equal). **Henri Thomassen:** Formal analysis (equal); Writing‐review & editing (equal). **Rita Triebskorn:** Conceptualization (equal); Data curation (equal); Investigation (equal); Writing‐review & editing (equal).

## Data Availability

All data and detailed information on the statistics can be obtained from Dryad under https://doi.org/10.5061/dryad.51c59zw5m or from the corresponding author upon request.

## References

[ece37002-bib-0001] Allen, J. A. (2004). Avian and mammalian predators of terrestrial gastropods In G. M. Barker (Ed.), Natural enemies of terrestrial molluscs (pp. 1–37). CABI Publishing.

[ece37002-bib-0002] Arad, Z. , Mizrahi, T. , Goldenberg, S. , & Heller, J. (2010). Natural annual cycle of heat shock protein expression in land snails: Desert versus Mediterranean species of *Sphincterochila* . Journal of Experimental Biology, 213(20), 3487–3495. 10.1242/jeb.047670 20889829

[ece37002-bib-0003] Bates, D. , Maechler, M. , Bolker, B. , & Walker, S. (2015). Fitting linear mixed‐effects models using lme4. Journal of Statistical Software, 67, 1–48. 10.18637/jss.v067.i01

[ece37002-bib-0004] Bond, A. B. (2007). The evolution of color polymorphism: Crypticity, searching images, and apostatic selection. Annual Reviews of Ecology, Evolution, and Systematics, 38, 489–514. 10.1146/annurev.ecolsys.38.091206.095728

[ece37002-bib-0005] Boyle, W. A. , Sandercock, B. K. , & Martin, K. (2015). Patterns and drivers of intraspecific variation in avian life history along elevational gradients: A meta‐analysis. Biological Reviews, 91, 469–482. 10.1111/brv.12180 25765584

[ece37002-bib-0006] Bradford, M. M. (1976). A rapid and sensitive method for the quantitation of microgram quantities of protein utilizing the principle of protein‐dye binding. Analytical Biochemistry, 72, 248–254. 10.1006/abio.1976.9999 942051

[ece37002-bib-0007] Brook, B. W. , Sodhi, N. S. , & Bradshaw, C. J. A. (2008). Synergies among extinction drivers under global change. Trends in Ecology & Evolution, 23, 453–460. 10.1016/j.tree.2008.03.011 18582986

[ece37002-bib-0008] Cahill, A. E. , Aiello‐Lammens, M. E. , Fisher‐Reid, M. C. , Hua, X. , Karanewsky, C. J. , Ryu, H. Y. , Sbeglia, G. C. , Spagnolo, F. , Waldron, J. B. , Warsi, O. , & Wiens, J. J. (2013). How does climate change cause extinction? Proceedings of the Royal Society B Biological Sciences, 280, 20121890 10.1098/rspb.2012.1890 PMC3574421 23075836

[ece37002-bib-0009] Cain, A. J. (1983). Ecology and ecogenetics of terrestrial molluscan populations In W. D. Russell‐Hunter (Ed.), The Mollusca, Vol. 6, Ecology (pp. 597–647). Academic Press.

[ece37002-bib-0010] Cain, A. J. (1984). Genetics of some morphs in the land snail *Theba pisana* . Malacologia, 25, 381–411.

[ece37002-bib-0011] Cain, A. J. , Cook, L. M. , & Currey, J. D. (1990). Population size and morph frequency in a long‐term study of *Cepaea nemoralis* . Proceedings of the Royal Society B Biological Sciences, 240, 231–250.

[ece37002-bib-0012] Cain, A. J. , & Currey, J. D. (1963). Area effects in *Cepaea* . Philosophical Transactions of the Royal Society B Biological Sciences, 246, 1–81. 10.1098/rstb.1963.0001

[ece37002-bib-0013] Cain, A. J. , & Sheppard, P. M. (1954). Natural selection in *Cepaea* . Genetics, 39, 89–116. 17247470 10.1093/genetics/39.1.89 PMC1209639

[ece37002-bib-0014] Cameron, R. A. D. (1992). Change and stability in *Cepaea* populations over 25 years – a case of climatic selection. Proceedings of the Royal Society London Biological Sciences, 248, 181–187. 10.1098/rspb.1992.0060

[ece37002-bib-0015] Cameron, R. A. D. (2001). *Cepaea nemoralis* in a hostile environment: Continuity, colonizations and morph frequencies over time. Biological Journal of the Linnean Society, 74, 255–264. 10.1111/j.1095-8312.2001.tb01390.x

[ece37002-bib-0016] Cameron, R. A. D. , & Cook, L. M. (2012). Habitat and the shell polymorphism of *Cepaea nemoralis* (L.): Interrogating the Evolution MegaLab database. Journal of Molluscan Studies, 78, 179–184. 10.1093/mollus/eyr052

[ece37002-bib-0017] Cameron, R. A. D. , Cook, L. M. , & Greenwood, J. J. D. (2013). Change and stability in a steep morph‐frequency cline in the snail *Cepaea nemoralis* (L.) over 43 years. Biological Journal of the Linnean Society, 108, 473–483. 10.1111/j.1095-8312.2012.02033.x

[ece37002-bib-0018] Cameron, R. A. D. , & Pokryszko, B. M. (2008). Variation in *Cepaea* populations over 42 years: Climatic fluctuations destroy a topographical relationship of morph frequencies. Biological Journal of the Linnean Society, 95, 53–61. 10.1111/j.1095-8312.2008.01042.x

[ece37002-bib-0019] Chapperon, C. , & Seuront, L. (2011). Behavioral thermoregulation in a tropical gastropod: Links to climate change scenarios. Global Change Biology, 17(4), 1740–1749. 10.1111/j.1365-2486.2010.02356.x

[ece37002-bib-0020] Chapperon, C. , & Seuront, L. (2012). Keeping warm in the cold: On the thermal benefits of aggregation behaviour in an intertidal ectotherm. Journal of Thermal Biology, 37(8), 640–647. 10.1016/j.jtherbio.2012.08.001

[ece37002-bib-0021] Clarke, B. C. (1969). The evidence for apostatic selection. Heredity, 24, 347–352. 10.1038/hdy.1969.52 5262945

[ece37002-bib-0022] Cook, L. M. (2005). Disequilibrium in some *Cepaea* populations. Heredity, 94, 497–500. 10.1038/sj.hdy.6800645 15742002

[ece37002-bib-0023] Cook, L. M. , Cowie, R. H. , & Jones, J. S. (1999). Change in morph frequency in the snail *Cepaea nemoralis* on the Marlborough Downs. Heredity, 82, 336–342. 10.1038/sj.hdy.6884920 10336709

[ece37002-bib-0024] Cook, L. M. , & Freeman, P. M. (1986). Heating properties of morphs of the mangrove snail *Littoraria pallescens* . Biological Journal of the Linnean Society, 29, 295–300. 10.1111/j.1095-8312.1986.tb00281.x

[ece37002-bib-0025] Cook, L. M. , & Pettitt, C. W. A. (1998). Morph frequencies in the snail *Cepaea nemoralis*: Changes with time and their interpretation. Biological Journal of the Linnean Society, 64, 137–150. 10.1111/j.1095-8312.1998.tb01538.x

[ece37002-bib-0026] Cowie, R. H. (1984). Ecogenetics of *Theba pisana* (Pulmonata: Helicidae) at the northern edge of its range. Malacologia, 25, 361–380.

[ece37002-bib-0027] Cowie, R. H. (1985). Microhabitat choice and high temperature tolerance in the land snail *Theba pisana* (Mollusca: Gastropoda). Journal of Zoology, 207, 201–211. 10.1111/j.1469-7998.1985.tb04924.x

[ece37002-bib-0028] Cowie, R. H. (1990). Climatic selection on body colour in the land snail *Theba pisana* (Pulmonata: Helicidae). Heredity, 65, 123–126. 10.1038/hdy.1990.78

[ece37002-bib-0029] Cowie, R. H. (1992). Shell pattern polymorphism in a 13‐year study of the land snail *Theba pisana* (Müller) (Pulmonata: Helicidae). Malacologia, 34, 87–97.

[ece37002-bib-0030] Cowie, R. H. , & Jones, J. S. (1985). Climatic selection on body colour in *Cepaea* . Heredity, 55, 261–267. 10.1038/hdy.1990.78

[ece37002-bib-0031] Cowie, R. H. , & Jones, J. S. (1998). Gene frequency changes in *Cepaea* snails on the Marlborough Downs over 25 years. Biological Journal of the Linnean Society, 65, 233–255. 10.1111/j.1095-8312.1998.tb01141.x

[ece37002-bib-0032] Cramer, W. , Guiot, J. , Fader, M. , Garrabou, J. , Gattuso, J.‐P. , Iglesias, A. , Lange, M. A. , Lionello, P. , Llasat, M. C. , Paz, S. , Peñuelas, J. , Snoussi, M. , Toreti, A. , Tsimplis, M. N. , & Xoplaki, E. (2018). Climate change and interconnected risks to sustainable development in the Mediterranean. Nature Climate Change, 8, 972–980. 10.1038/s41558-018-0299-2

[ece37002-bib-0033] Currey, J. D. , & Cain, A. J. (1968). Climate and selection of banding morphs in *Cepaea* from the climatic optimum to the present day. Philosophical Transactions of the Royal Society of London B Biological Sciences, 253, 483–498. 10.1098/rstb.1968.0008

[ece37002-bib-0034] Debecker, S. , & Stoks, R. (2019). Pace of life syndrome under warming and pollution: Integrating life history, behavior, and physiology across latitudes. Ecological Monographs, 89(1), e01332 10.1002/ecm.1332

[ece37002-bib-0035] Di Lellis, M. A. , Seifan, M. , Troschinski, S. , Mazzia, C. , Capowiez, Y. , Triebskorn, R. , & Köhler, H.‐R. (2012). Solar radiation stress in climbing snails: Behavioural and intrinsic features define the Hsp70 level in natural populations of *Xeropicta derbentina* (Pulmonata). Cell Stress and Chaperones, 17(6), 717–727. 10.1007/s12192-012-0344-4 22639082 PMC3468672

[ece37002-bib-0036] Di Lellis, M. A. , Sereda, S. , Geißler, A. , Picot, A. , Arnold, P. , Lang, S. , Troschinski, S. , Dieterich, A. , Hauffe, T. , Capowiez, Y. , Mazzia, C. , Knigge, T. , Monsinjon, T. , Krais, S. , Wilke, T. , Triebskorn, R. , & Köhler, H.‐R. (2014). Phenotypic diversity, population structure, and stress protein‐based capacitoring in populations of *Xeropicta derbentina*, a heat‐tolerant land snail species. Cell Stress and Chaperones, 19(6), 791–800. 10.1007/s12192-014-0503-x 24609822 PMC4389839

[ece37002-bib-0037] Dieterich, A. , Fischbach, U. , Ludwig, M. , Di Lellis, M. A. , Troschinski, S. , Gärtner, U. , Triebskorn, R. , & Köhler, H.‐R. (2013). Daily and seasonal changes in heat exposure and the Hsp70 level of individuals from a field population of *Xeropicta derbentina* (Krynicki 1836) (Pulmonata, Hygromiidae) in Southern France. Cell Stress and Chaperones, 18(4), 405–414. 10.1007/s12192-012-0393-8 23250584 PMC3682011

[ece37002-bib-0038] Dieterich, A. , Troschinski, S. , Schwarz, S. , Di Lellis, M. A. , Henneberg, A. , Fischbach, U. , Ludwig, M. , Gärtner, U. , Triebskorn, R. , & Köhler, H.‐R. (2015). Hsp70 and lipid peroxide levels following heat stress in *Xeropicta derbentina* (Krynicki 1836) (Gastropoda, Pulmonata) with regard to different colour morphs. Cell Stress and Chaperones, 20, 159–168. 10.1007/s12192-014-0534-3 25108358 PMC4255243

[ece37002-bib-0039] Dowd, W. W. , King, F. A. , & Denny, M. W. (2015). Thermal variation, thermal extremes and the physiological performance of individuals. Journal of Experimental Biology, 218, 1956–1967. 10.1242/jeb.114926 26085672

[ece37002-bib-0040] Eckwert, H. , Alberti, G. , & Köhler, H.‐R. (1997). The induction of stress proteins (hsp) in Oniscus asellus (Isopoda) as a molecular marker of multiple heavy metal exposure. I. Principles and Toxicological Assessment. Ecotoxicology, 6(5), 249–262. 10.1023/A:1018682928839

[ece37002-bib-0041] Endler, J. A. (1978). A predator’s view of animals colour patterns. Evolutionary Biology, 11, 319–364. 10.1007/978-1-4615-6956-5_5

[ece37002-bib-0042] European Environment Agency (2018). Global and European temperature (CSI 012, CLIM 001). Retrieved from https://www.eea.europa.eu/data-and-maps/indicators/global-and-european-temperature-8/assessment

[ece37002-bib-0043] Exposito‐Alonso, M. , 500 Genomes Field Experiment Team , Burbano, H. A. , Bossdorf, O. , Nielsen, R. , & Weigel, D. (2019). Natural selection on the *Arabidopsis thaliana* genome in present and future climates. Nature, 573, 126–129. 10.1038/s41586-019-1520-9 31462776

[ece37002-bib-0044] Gaitán‐Espitia, J. D. , Belen, A. M. , Lardies, M. A. , & Nespolo, R. F. (2013). Variation in thermal sensitivity and thermal tolerances in an invasive species across a climatic gradient: Lessons from the land snail *Cornu aspersum* . PLoS One, 8, e70662 10.1371/journal.pone.0070662 23940617 PMC3734266

[ece37002-bib-0045] Gaschler, M. M. , & Stockwell, B. R. (2017). Lipid peroxidation in cell death. Biochemical and Biophysical Research Communications, 482(3), 419–425. 10.1016/j.bbrc.2016.10.086 28212725 PMC5319403

[ece37002-bib-0046] Gerken, A. R. , Eller, O. C. , Hahn, D. A. , & Morgan, T. J. (2015). Constraints, independence, and evolution of thermal plasticity: Probing genetic architecture of long‐ and short‐term thermal acclimation. Proceedings of the Natural Academy of Sciences of the USA, 112, 4399–4404. 10.1073/pnas.1503456112 PMC4394312 25805817

[ece37002-bib-0047] Gerlach, J. (2007). Short‐term climate change and the extinction of the snail *Rhachistia aldabrae* (Gastropoda: Pulmonata). Biology Letters, 3, 581–584. 10.1098/rsbl.2007.0316 17666376 PMC2391199

[ece37002-bib-0048] Giorgi, F. , & Lionelli, P. (2008). Climate change projections for the Mediterranean region. Global and Planetary Change, 63(2–3), 90–104. 10.1016/j.gloplacha.2007.09.005

[ece37002-bib-0049] Hazel, W. N. , & Johnson, M. S. (1990). Microhabitat choice and polymorphism in the land snail Theba pisana (Müller). Heredity, 65, 449–454. 10.1038/hdy.1990.116

[ece37002-bib-0050] Heath, D. J. (1975). Colour, sunlight and internal temperatures in the land‐snail Cepaea nemoralis (L.). Oecologia, 19, 29–38. 10.1007/BF00377587 28308828

[ece37002-bib-0051] Heller, J. (1981). Visual versus climatic selection of shell banding in the landsnail *Theba pisana* in Israel. Journal of Zoology, 194, 85–101. 10.1111/j.1469-7998.1981.tb04580.x

[ece37002-bib-0052] Heller, J. , & Gadot, M. (1984). Shell polymorphism of *Theba pisana* – the effects of rodent distribution. Malacologia, 25, 349–354.

[ece37002-bib-0053] Henry, G. H. R. , & Molau, U. (1997). Tundra plants and climate change: The International Tundra Experiment (ITEX). Global Change Biology, 3(Suppl. 1), 1–9. 10.1111/j.1365-2486.1997.gcb132.x

[ece37002-bib-0054] Hermes‐Lima, M. , Willmore, W. G. , & Storey, K. B. (1995). Quantification of lipid peroxidation in tissue extracts based on Fe(III)xylenol orange complex formation. Free Radical Biology and Medicine, 19, 271–280. 10.1016/0891-5849(95)00020-x 7557541

[ece37002-bib-0055] Hill, A. B. (1965). The environment and disease: Association or causation? Proceedings of the Royal Society of Medicine, 58(5), 295–300. 10.1177/003591576505800503 14283879 PMC1898525

[ece37002-bib-0056] Jacob, D. , Petersen, J. , Eggert, B. , Alias, A. , Christensen, O. B. , Bouwer, L. M. , Braun, A. , Colette, A. , Déqué, M. , Georgievski, G. , Georgopoulou, E. , Gobiet, A. , Menut, L. , Nikulin, G. , Haensler, A. , Hempelmann, N. , Jones, C. , Keuler, K. , Kovats, S. , … Yiou, P. (2014). EURO‐CORDEX: New high‐resolution climate change projections for European impact research. Regional Environmental Change, 14(2), 563–578. 10.1007/s10113-013-0499-2

[ece37002-bib-0057] Johnson, M. S. (1980). Association and shell banding and habitat in a colony of the land snail *Theba pisana* . Heredity, 45, 7–14. 10.1038/hdy.1980.46

[ece37002-bib-0058] Johnson, M. S. (2011). Thirty‐four years of climatic selection in the land snail *Theba pisana* . Heredity, 106, 741–748. 10.1038/hdy.2010.114 20823904 PMC3186227

[ece37002-bib-0059] Johnson, M. S. (2012). Epistasis, phenotypic disequilibrium and contrasting associations with climate in the land snail *Theba pisana* . Heredity, 108(3), 229–235. 10.1038/hdy.2011.62 21811302 PMC3282386

[ece37002-bib-0060] Jones, J. S. (1973). Ecological genetics and natural selection in Molluscs. Science, 182, 546–552. 10.1126/science.182.4112.546 4746485

[ece37002-bib-0061] Jones, J. S. , Leith, B. H. , & Rawlings, P. (1977). Polymorphism in *Cepaea*: A problem with too many solutions? Annual Review of Ecological Systems, 8, 109–143. 10.1146/annurev.es.08.110177.000545

[ece37002-bib-0062] Kerstes, N. A. G. , Breeschoten, T. , Kalkman, V. J. , & Schilthuizen, M. (2019). Snail shell colour evolution in urban heat islands detected via citizen science. Communications Biology, 2, 264 10.1038/s42003-019-0511-6 31341963 PMC6642149

[ece37002-bib-0063] Killen, S. S. , Marras, S. , Metcalfe, N. B. , McKenzie, D. J. , & Domenici, P. (2013). Environmental stressors alter relationships between physiology and behaviour. Trends in Ecology & Evolution, 28, 651–658. 10.1016/j.tree.2013.05.005 23756106

[ece37002-bib-0064] Knigge, T. , Di Lellis, M. A. , Monsinjon, T. , & Köhler, H.‐R. (2017). Relevance of body size and shell colouration for thermal absorption and heat loss in white garden snails, *Theba pisana* (Helicidae), from Northern France. Journal of Thermal Biology, 69, 54–63. 10.1016/j.jtherbio.2017.06.001 29037405

[ece37002-bib-0065] Köhler, H.‐R. , Lazzara, R. , Dittbrenner, N. , Capowiez, Y. , Mazzia, C. , & Triebskorn, R. (2009). Snail phenotypic variation and stress proteins: Do different heat response strategies contribute to Waddington's widget in field populations? Journal of Experimental Zoology Part B: Molecular and Developmental Evolution, 312B(2), 136–147. 10.1002/jez.b.21253 19065565

[ece37002-bib-0066] Köhler, H.‐R. , Schultz, C. , Scheil, A. E. , Triebskorn, R. , Seifan, M. , & Di Lellis, M. A. (2013). Historic data analysis reveals ambient temperature as a source of phenotypic variation in populations of the land snail *Theba pisana* . Biological Journal of the Linnean Society, 109(1), 241–256. 10.1111/bij.12035

[ece37002-bib-0067] Kotsakiozi, P. , Parmakelis, A. , Aggeli, I.‐K. , Gaitanaki, C. , Giokas, G. , & Valakos, E. D. (2015). Water balance and expression of heat‐shock protein 70 in *Codringtonia* species: A study within a phylogenetic framework. Journal of Molluscan Studies, 81, 24–36. 10.1093/mollus/eyu042

[ece37002-bib-0068] Lenth, R. (2018). emmeans: Estimated Marginal Means, aka Least‐Squares Means. R package version 1.2.2. Retrieved from https://CRAN.R-project.org/package=emmeans

[ece37002-bib-0069] Marion, G. M. , Henry, G. H. R. , Freckman, D. W. , Johnstone, J. , Jones, G. , Jones, M. H. , Levesque, E. , Molau, U. , Molgaard, P. , Parsons, A. N. , Svoboda, J. , & Virginia, R. A. (1997). Open‐top designs for manipulating field temperature in high‐latitude ecosystems. Global Change Biology, 3(Suppl. 1), 20–32. 10.1111/j.1365-2486.1997.gcb136.x

[ece37002-bib-0070] Mariotti, A. , Pan, Y. , Zeng, N. , & Alessandri, A. (2015). Long‐term climate change in the Mediterranean region in the midst of decadal variability. Climate Dynamics, 44, 1437–1456. 10.1007/s00382-015-2487-3

[ece37002-bib-0071] McQuaid, C. D. , Branch, G. M. , & Frost, P. G. H. (1979). Aestivation behaviour and thermal relations of the pulmonate *Theba pisana* in a semi‐arid environment. Journal of Thermal Biology, 4(1), 47–55. 10.1016/0306-4565(79)90045-7

[ece37002-bib-0072] Mizrahi, T. , Goldenberg, S. , Heller, J. , & Arad, Z. (2015). Natural variation in resistance to desiccation and heat shock protein expression in the land snail *Theba pisana* along a climatic gradient. Physiological and Biochemical Zoology, 88, 66–80. 10.1086/679485 25590594

[ece37002-bib-0073] Mizrahi, T. , Goldenberg, S. , Heller, J. , & Arad, Z. (2016). Geographic variation in thermal tolerance and strategies of heat shock protein expression in the land snail *Theba pisana* in relation to genetic structure. Cell Stress and Chaperones, 21, 219–238. 10.1007/s12192-015-0652-6 26503612 PMC4786534

[ece37002-bib-0074] Mizrahi, T. , Heller, J. , Goldenberg, S. , & Arad, Z. (2010). Heat shock proteins and resistance to desiccation in congeneric land snails. Cell Stress and Chaperones, 15(4), 351–363. 10.1007/s12192-009-0150-9 19953352 PMC3082649

[ece37002-bib-0075] Mizrahi, T. , Heller, J. , Goldenberg, S. , & Arad, Z. (2011). Heat shock protein expression in relation to the reproductive cycle in land snails: Implications for survival. Comparative Biochemistry and Physiology A, 160, 149–155. 10.1007/s12192-012-0341-7 21664480

[ece37002-bib-0076] Mizrahi, T. , Heller, J. , Goldenberg, S. , & Arad, Z. (2012a). The heat shock response in congeneric land snails (*Sphincterochila*) from different habitats. Cell Stress and Chaperones, 17, 639–645. 10.1007/s12192-012-0340-8 22535471 PMC3535165

[ece37002-bib-0077] Mizrahi, T. , Heller, J. , Goldenberg, S. , & Arad, Z. (2012b). Heat shock proteins and survival strategies in congeneric land snails (*Sphincterochila*) from different habitats. Cell Stress and Chaperones, 17, 523–527. 10.1007/s12192-012-0340-8 22528052 PMC3535171

[ece37002-bib-0078] Moreno‐Rueda, G. (2008). The color white diminishes weight loss during aestivation in the arid‐dwelling land snail *Sphincterochila (Albea) candidissima* . Iberus, 26, 47–51.

[ece37002-bib-0079] Moreno‐Rueda, G. (2009). Disruptive selection by predation offsets stabilizing selection on shell morphology in the land snail *Iberus g. gualtieranus* . Evolutionary Ecology, 23, 463–471. 10.1007/s10682-008-9245-5

[ece37002-bib-0080] Murray, J. , & Clarke, B. (1978). Change of gene frequency in *Cepaea nemoralis* over fifty years. Malacologia, 17, 317–330.

[ece37002-bib-0081] Ng, T. P. T. , Lau, S. L. Y. , Seuront, L. , Davies, M. S. , Stafford, R. , Marshall, D. J. , & Williams, G. A. (2017). Linking behaviour and climate change in intertidal ectotherms: Insights from littorinid snails. Journal of Experimental Marine Biology and Ecology, 492, 121–131. 10.1016/j.jembe.2017.01.023

[ece37002-bib-0082] Ożgo, M. (2011). Rapid evolution in unstable habitats: A success story of the polymorphic land snail *Cepaea nemoralis* (Gastropoda: Pulmonata). Biological Journal of the Linnean Society, 102, 251–262. 10.1111/j.1095-8312.2010.01585.x

[ece37002-bib-0083] Ożgo, M. (2014). Rapid evolution and the potential for evolutionary rescue in land snails. Journal of Molluscan Studies, 80, 286–290. 10.1093/mollus/eyu029

[ece37002-bib-0084] Ożgo, M. , & Bogucki, Z. (2011). Colonization, stability, and adaptation in a transplant experiment of the polymorphic land snail *Cepaea nemoralis* (Gastropoda: Pulmonata) at the edge of its geographical range. Biological Journal of the Linnean Society, 104, 462–470. 10.1111/j.1095-8312.2011.01732.x

[ece37002-bib-0085] Ożgo, M. , & Kinnison, M. T. (2008). Contigency and determinism during convergent contemporary evolution in the polymorphic land snail *Cepaea nemoralis* . Evolutionary Ecology Research, 10, 721–733.

[ece37002-bib-0086] Ożgo, M. , & Komorowska, A. (2009). Shell banding polymorphism in *Cepaea vindobonensis* in relation to habitat in southeastern Poland. Malacologia, 51, 81–88. 10.4002/040.051.0105

[ece37002-bib-0087] Ożgo, M. , & Schilthuizen, M. (2011). Evolutionary change in *Cepaea nemoralis* shell colour over 43 years. Global Change Biology, 18, 74–81. 10.1111/j.1365-2486.2011.02514.x

[ece37002-bib-0088] Pörtner, H. O. (2002). Climate variations and the physiological basis of temperature dependent biogeography: Systemic to molecular hierarchy of thermal tolerance in animals. Comparative Biochemistry and Physiology A, 132, 739–761. 10.1016/s1095-6433(02)00045-4 12095860

[ece37002-bib-0089] Punzalan, D. , Rodd, F. H. , & Hughes, K. A. (2005). Perceptual processes and the maintenance of polymorphism through frequency‐dependent predation. Evolutionary Ecology, 19, 303–320. 10.1007/s10682-005-2777-z

[ece37002-bib-0090] R Development Core Team (2014). R: A language and environment for statistical computing. : Computing RFfS.

[ece37002-bib-0091] Reuner, A. , Brümmer, F. , & Schill, R. O. (2008). Heat shock proteins (Hsp70) and water content in the estivating Mediterranean Grunt Snail (*Cantareus apertus*). Comparative Biochemistry and Physiology Part B Biochemistry and Molecular Biology, 151, 28–31. 10.1016/j.cbpb.2008.05.004 18579425

[ece37002-bib-0092] Richards, A. V. , & Murray, J. J. (1975). The relation of phenotype to habitat in an introduced colony of *Cepaea nemoralis* . Heredity, 34, 128–131. 10.1038/hdy.1975.13 1054673

[ece37002-bib-0093] Riddle, W. A. (1990). High temperature tolerance in three species of land snails. Journal of Thermal Biology, 15(2), 119–124. 10.1016/0306-4565(90)90028-G

[ece37002-bib-0094] Rosin, Z. M. , Olborska, P. , Surmacki, A. , & Tryjanowski, P. (2011). Differences in predatory pressure on terrestrial snails by birds and mammals. Journal of Biosciences, 36, 691–699. 10.1007/s12038-011-9077-2 21857115

[ece37002-bib-0095] Scheil, A. E. , Köhler, H.‐R. , & Triebskorn, R. (2011). Heat tolerance and recovery in Mediterranean land snails after pre‐exposure in the field. Journal of Molluscan Studies, 77(2), 165–174. 10.1093/mollus/eyr003

[ece37002-bib-0096] Schilthuizen, M. (2012). Scrutinising snail shells. Heredity, 108, 364–365. 10.1038/hdy.2011.88 21934704 PMC3313051

[ece37002-bib-0097] Schilthuizen, M. (2013). Rapid, habitat‐related evolution of land snail colour morphs on reclaimed land. Heredity, 110, 247–252. 10.1038/hdy.2012.74 23149460 PMC3669759

[ece37002-bib-0098] Schmidt‐Nielsen, K. , Taylor, C. R. , & Shkolnik, A. (1971). Desert snails: Problems of heat, water and food. Journal of Experimental Biology, 55(2), 385–398. 10.1242/jeb.55.2.385 5114030

[ece37002-bib-0099] Schweizer, M. , Triebskorn, R. , & Köhler, H.‐R. (2019). Snails in the sun: Strategies of terrestrial gastropods to cope with hot and dry conditions. Ecology & Evolution, 9, 12940–12960. 10.1002/ece3.5607 31788227 PMC6875674

[ece37002-bib-0100] Silvertown, J. , Cook, L. , Cameron, R. , Dodd, M. , McConway, K. , Worthington, J. , Skelton, P. , Anton, C. , Bossdorf, O. , Baur, B. , Schilthuizen, M. , Fontaine, B. , Sattmann, H. , Bertorelle, G. , Correia, M. , Oliveira, C. , Pokryszko, B. , Ożgo, M. , Stalažs, A. , … Juan, X. (2011). Citizen science reveals unexpected continental‐scale evolutionary change in a model organism. PLoS One, 6(4), e18927 10.5061/dryad.p7h802r0 21556137 PMC3083392

[ece37002-bib-0101] Staikou, A. E. (1999). Shell temperature, activity and resistance to desiccation in the polymorphic land snail *Cepaea vindobonensis* . Journal of Molluscan Studies, 65, 171–184. 10.1046/j.1365-2435.2001.00525.x

[ece37002-bib-0102] Stevens, V. M. , Trochet, A. , Blanchet, S. , Moulherat, S. , Clobert, J. , & Baguette, M. (2013). Dispersal syndromes and the use of life‐histories to predict dispersal. Evolutionary Applications, 6, 630–642. 10.1111/eva.12049 23789030 PMC3684744

[ece37002-bib-0103] Stine, O. C. (1989). *Cepaea nemoralis* from Lexington, Virginia: The isolation and characterization of their mitochondrial DNA, the implications for their origin and climatic selection. Malacologia, 30, 305–315.

[ece37002-bib-0104] Triebskorn, R. , Adam, S. , Behrens, A. , Beier, S. , Böhmer, J. , Braunbeck, T. , Casper, H. , Dietze, U. , Gernhöfer, M. , Honnen, W. , Köhler, H.‐R. , Körner, W. , Konradt, J. , Lehmann, R. , Luckenbach, T. , Oberemm, A. , Schwaiger, J. , Segner, H. , Strmac, M. , … Traunspurger, W. (2003). Establishing causality between pollution and effects at different levels of biological organization: The VALIMAR project. Human and Ecological Risk Assessment, 9(1), 171–194. 10.1080/713609858

[ece37002-bib-0105] Troschinski, S. , Di Lellis, M. A. , Sereda, S. , Hauffe, T. , Wilke, T. , Triebskorn, R. , & Köhler, H.‐R. (2014). Intraspecific variation in cellular and biochemical heat response strategies of Mediterranean *Xeropicta derbentina* [Pulmonata, Hygromiidae]. PLoS One, 9(1), e86613 10.1371/journal.pone.0086613 24475158 PMC3903566

[ece37002-bib-0106] Troschinski, S. , Dieterich, A. , Krais, S. , Triebskorn, R. , & Köhler, H.‐R. (2014). Antioxidant defence and stress protein induction following heat stress in the Mediterranean snail *Xeropicta derbentina* . Journal of Experimental Biology, 217, 4399–4405. 10.1242/jeb.113167 25394630

[ece37002-bib-0107] Tull, T. , Schmidt, D. , & Köhler, H.‐R. (2020). Role of ambient wavelength, shell size and pigmentation intensity in the heating of Mediterranean *Theba pisana* (Eupulmonata: Helicidae). Journal of Molluscan Studies, 86(3), 249–253. 10.1093/mollus/eyaa006

[ece37002-bib-0108] Wall, S. , Carter, M. A. , & Clarke, B. (1980). Temporal changes of gene frequencies in *Cepaea hortensis* . Biological Journal of the Linnean Society, 14, 303–317. 10.1111/j.1095-8312.1980.tb00111.x

[ece37002-bib-0109] Welshofer, K. B. , Zarnetske, P. L. , Lany, N. K. , & Thompson, L. A. E. (2018). Open‐top chambers for temperature manipulation in taller‐stature plant communities. Methods in Ecology and Evolution, 9(2), 254–259. 10.1111/2041-210X.12863

[ece37002-bib-0110] Yan, X. M. , Ni, Z. , Chang, L. , Wang, K. H. , & Wu, D. H. (2015). Soil warming elevates the abundance of collembola in the Songnen Plain of China. Sustainability, 7(2), 1161–1171. 10.3390/su7021161

